# Costs and benefits of phytoplankton motility

**Published:** 2025-08-09

**Authors:** Peyman Fahimi, Andrew J. Irwin, Michael Lynch

**Affiliations:** (a) Department of Mathematics & Statistics, Dalhousie University, Halifax, NS B3H4R2 Canada.; (b) Center for Mechanisms of Evolution, Biodesign Institute, Arizona State University, Tempe, AZ 85287 USA.

**Keywords:** energetic cost, motility benefit, phytoplankton, sinking speed, swimming speed

## Abstract

The motility skills of phytoplankton have evolved and persisted over millions of years, primarily in response to factors such as nutrient and light availability, temperature and viscosity gradients, turbulence, and predation pressure. Phytoplankton motility is broadly categorized into swimming and buoyancy regulation. Despite studies in the literature exploring the motility costs and benefits of phytoplankton, there remains a gap in our integrative understanding of direct and indirect energy expenditures, starting from when an organism initiates movement due to any biophysical motive, to when the organism encounters intracellular and environmental challenges. Here we gather available pieces of this puzzle from literature in biology, physics, and oceanography to paint an overarching picture of our current knowledge. The characterization of sinking and rising behavior as passive motility has resulted in the concept of sinking and rising internal efficiency being overlooked. We define this efficiency based on any energy dissipation associated with processes of mass density adjustment, as exemplified in structures like frustules and vacuoles. We propose that sinking and rising are active motility processes involving non-visible mechanisms, as species demonstrate active and rapid strategies in response to turbulence, predation risk, and gradients of nutrients, light, temperature, and viscosity. Identifying intracellular buoyancy-regulating dissipative processes offers deeper insight into the motility costs relative to the organism's total metabolic rate.

## 1. Introduction

Phytoplankton, organisms capable of photosynthesis and mixotrophy, play pivotal roles in carbon cycling and primary production within the ocean ecosystem (Finkel et al., 2010). Investigating their locomotion - both swimming and sinking/rising - provides insights into marine and freshwater ecology, including biogeochemical cycling (Kriest & Oschlies, 2008), dynamics of food webs (Michaels & Silver, 1988), biological carbon pumps (Giering et al., 2020), population growth rates (Huisman & Sommeijer, 2002), and phenomena like marine snow and blooming (Turner, 2002). Phytoplankton motility primarily enables vertical movement in the water column, allowing cells to access light near the surface and nutrients at depth. This repositioning ability can enhance growth and reproduction, potentially triggering bloom formation under favorable environmental conditions. Motile phytoplankton can also avoid nutrient-depleted layers and, in some cases, reduce exposure to grazers, thereby influencing population dynamics and trophic interactions. Some species regulate their buoyancy or form sinking aggregates, facilitating the downward transport of organic matter and nutrients. This vertical export is a core process in the biological carbon pump, contributing to the sequestration of surface carbon in the deep ocean and supporting the formation of marine snow, aggregates that drive vertical fluxes of organic material and shape global biogeochemical cycles.

In this review, we delve into the costs and benefits associated with the locomotion of phytoplankton species. Specifically, we explore their swimming and buoyancy regulation over short distances in patchy aquatic environments and over long distances as part of migratory behaviors. Our examination encompasses both biological and physical dimensions of this phenomenon of oceanography shedding light on the gaps and potential future research directions.

Before evaluating the costs and benefits of phytoplankton motility, it is essential to identify which species are most significant in terms of both their abundance and their contribution to total primary production - an assessment that varies by geographical location. For instance, during the boreal winter of 2017 along a transect through the North Pacific Subtropical Gyre and the California Current System, the three most abundant groups were coccolithophores (35.5%), diatoms (25.2%), and dinoflagellates (19.5%) (Matek et al., 2023). However, a global-scale dataset spanning 2002–2022 indicates a different abundance ranking: chlorophytes (e.g., green algae) (~26%) are most prevalent, followed by diatoms (~24%), haptophytes (e.g., coccolithophores) (~15%), cryptophytes (~10%), cyanobacteria (~8%), and dinoflagellates (~3%) (Li et al., 2023). When considering primary production globally (1998–2011), diatoms contribute about 50% of total phytoplankton production, while coccolithophores and chlorophytes each contribute roughly 20%, and cyanobacteria around 10% (Rousseaux & Gregg, 2013).

Swimming or floating in water is energetically costly for microorganisms because they operate at low Reynolds numbers due to their small size and slow speed. In this regime, inertial forces are negligible compared to dominant viscous forces, causing water to behave like a thick, sticky fluid - more like honey than a free-flowing liquid. As a result, even the smallest movement requires a constant energy input; if the organism stops pushing, it stops almost immediately. For example, a tiny microorganism moving at about 30 microns per second will barely continue moving if the propelling force suddenly stops - traveling only about 0.1 angstrom before coming to a halt. This deceleration occurs in just 0.6 microseconds (Purcell, 1977).

There are notable differences between swimming and sinking/rising, resulting in varying cost-benefit analyses across species. For example, diatoms demonstrate sinking and rising behaviors, whereas dinoflagellates possess the capability for both swimming and buoyancy regulation. This differentiation might address certain unresolved issues in the literature concerning variations in population growth rates among different phyla. For example, why do dinoflagellates typically exhibit lower population growth rates compared to diatoms of similar size, despite having higher protein, chlorophyll, and carbohydrate content (Hitchcock, 1982; Tang, 1996)? The deeper question is why an organism would want to do this, as selection puts a primacy on offspring production. Presumably, what is lost in growth rate is compensated by higher survival, i.e., avoidance of predators?

It has been suggested that dinoflagellates coexist with other phytoplankton species by using their swimming abilities to counteract physiological disadvantages (Ross & Sharples, 2007). However, does the benefit of swimming always outweigh its costs? In this study, through an extensive analysis of swimming costs, we demonstrate that these costs can be remarkably high relative to the total metabolic rate of the organism, even under conditions favorable for active growth. The significant contribution of such high metabolic costs may compel the organism to allocate less energy to growth, resulting in relatively lower population growth rates.

**Figure 1:**
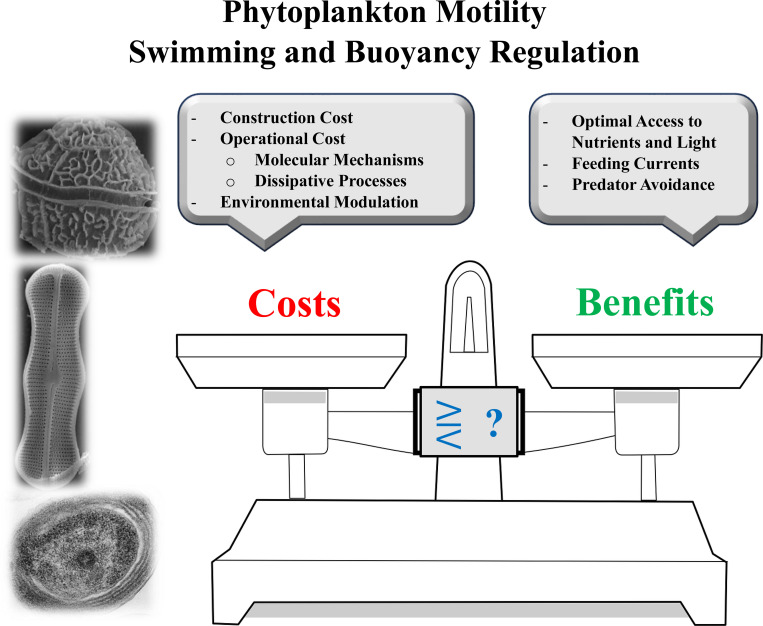
A conceptual depiction of the trade-off between the costs and benefits of phytoplankton motility in both swimmers and buoyancy regulators. The major components contributing to this trade-off are highlighted in two boxes. On the left, representative phytoplankton species are shown from top to bottom: a dinoflagellate, a diatom, and a cyanobacterium. Images are sourced from the public domain via Wikipedia.

This review follows a logical framework: to estimate motility costs and benefits, one must consider four key components (Figure 1):

1. **Construction Costs:** The first step is to examine the energetic costs of building the motility machinery, which includes both intracellular components and extracellular, surface-related structures - hereafter referred to as internal and external machinery, respectively. These construction costs can be either one-time investments or ongoing costs per unit time. They are typically quantified in terms of ATP molecules (the energy currency of living organisms) or standard energy units such as joules. To evaluate their significance, these costs can be compared to the organism’s total chemical energy content, including its protein, lipid, and carbohydrate reserves, yielding a percentage estimate of investment in motility.
2. **Operational Costs:** The second component involves assessing the energy required to operate the internal and external motility machinery. This is more complex than the construction cost, as it must account not only for the ATP demands of molecular processes driving motility but also for energy losses due to dissipation (i.e., heat produced by inefficient systems). These operational costs per unit time should be compared to the organism’s total metabolic rate - specifically, its total ATP production rate, which in phytoplankton primarily arises from mitochondrial and chloroplast activity under aerobic conditions.
3. **Environmental Modulation:** Finally, one must examine how environmental parameters - such as temperature, light, nutrient availability, viscosity, turbulence, viruses, and predators - affect motility costs, both directly and indirectly. This is arguably the most complex aspect of the problem, as environmental influences are multifaceted and difficult to quantify precisely.
4. **Benefits:** Assessing the benefits of motility primarily involves determining how - and to what extent - it enhances nutrient uptake, access to optimal light conditions, and predator avoidance. However, these benefits are strongly influenced by environmental conditions and geographical location, which shape nutrient and light availability as well as predation pressure.

The dissipative costs of motility machinery determine motility efficiency, which can be divided into external and internal efficiency. Motility external efficiency is influenced by cell surface structures, including the geometric shape of the cell body (Padisák, Soróczki-Pintér & Rezner, 2003; Nasouri, Vilfan & Golestanian, 2021) and the external features of motility-related structures, such as flagella (e.g., their length, number, geometry, angle, beat frequency, beat amplitude, and arrangement) (Purcell, 1977, 1997; Tam & Hosoi, 2011; Barsanti et al., 2016). In contrast, motility internal efficiency is governed by dissipative processes within intracellular structures, such as the axoneme (Spagnolie & Lauga, 2010; Chen et al., 2015), molecular motors, type IV pili, frustule, vacuole, gas vesicle, and coccolith.

Because swimming and buoyancy regulation arise from distinct intracellular mechanisms, their associated costs also differ. Swimmers, such as flagellated phytoplankton, invest in constructing and operating flagella (Schavemaker & Lynch, 2022), whereas in sinkers like diatoms, differences in mass density between cellular components (e.g., vacuoles) and structural compartments such as the frustule play significant roles in buoyancy regulation. This regulation is achieved through processes like substituting heavy ions with lighter ones (and vice versa) within vacuoles, or by modifying the composition and structure of the frustule (Miklasz & Denny, 2010; Raven & Lavoie, 2021).

Because buoyancy regulating species are frequently likened to passive particles in the literature, the assessment of energy dissipation linked to intracellular machineries has been neglected. Our perspective is that sinking and rising are active mechanisms for motility, using non-visible machinery. In this paper, we discuss the biological mechanisms involved in sinking internal efficiency, while the underlying physics needs to be formulated in future studies. The emphasis on non-visible motility mechanisms stems from the fact that determining the structure and function of visible motility machinery (such as flagella) is relatively easier with video microscopy, making it more accessible for researchers to investigate its efficiency. In contrast, studying non-visible motility mechanisms, such as those mediated by buoyancy-regulating systems like the frustule, vacuole, gas vesicle, and coccolith - found in diatoms, cyanobacteria, coccolithophores, and others - is more challenging.

Both slow and fast sinkers and swimmers exhibit vertical migratory behaviors (Wirtz et al., 2022). Slow movers engage in continuous migration over days and week, while faster ones undergo diel migration (Wirtz et al., 2022). Being fast or slow is a relative concept, enabling species to migrate between a specific depth and the ocean surface, or vice versa, within a defined timeframe, such as within a single day. Through migration, phytoplankton encounter changing environmental conditions across multiple parameters. The cost of motility is highly dependent on environmental variations, including gradients of nutrients (Cullen, 2015), light (Cullen, 2015), temperature (Kamykowski & Mccollum, 1986; Beveridge, Petchey & Humphries, 2010; Cullen, 2015), and viscosity (Guadayol et al., 2021), as well as on potentially chaotic trajectories (Marques da Silva, Duarte & Utkin, 2020) and events such as turbulence (Sengupta, Carrara & Stocker, 2017), predation (Kiørboe, 2024), and viral threats (Olive et al., 2022). These environmental parameters can influence motility costs both directly and indirectly.

Temperature can directly affect motility costs, as described by Stokes’ law, by altering environmental viscosity (Escobar et al., 2016), organismal size (Atkinson, Ciotti & Montagnes, 2003), and motility speed (Kamykowski & Mccollum, 1986). Temperature can indirectly influence cell shape by affecting surface tension (Lee, Kim & Needham, 2001), membrane elasticity, and permeability (Kirsch & Böckmann, 2019) - all of which impact motility external efficiency, which is partly shape-dependent. Temperature can also impair the functionality of motility structures such as flagella (Huang, Rifkin & Luck, 1977). As another example, environmental factors that reduce an organism’s overall metabolic rate - such as nutrient limitation, low temperatures, or anoxic conditions - can make motility disproportionately expensive relative to the total metabolic budget.

In regions with high viscosity gradients, the costs and benefits of hydrodynamic interactions may vary for swimmers and sinkers. In such environments, species may encounter elevated local micro-viscosities resulting from the combined biological activities of numerous other species, such as those found within marine aggregates, associated with extracellular polymeric substances and lysis (Guadayol et al., 2021), webs of colloids, and mucus sheets (Azam & Malfatti, 2007; Stocker, 2012). These conditions lead to the formation of heterogeneous viscosity gradients, which in turn affect the local distribution of species and the availability of nutrients (Guadayol et al., 2021). The viscosity gradients arising from hydrodynamic interactions, together with electrostatic interactions from larger particles and species, can potentially trap smaller particles and species. This creates a hitchhiking-like effect, where the smaller particles or species are carried along with the larger ones, influencing their distribution and behavior within the environment. In conclusion, viscosity is not constant; it varies across regions, which in turn can potentially alter motility costs. Typically, organisms are expected to slow down in higher viscosities, potentially leading to their accumulation in a region (Guadayol et al., 2021). However, reports indicate that *Chlamydomonas reinhardtii* employs tactics to reorient itself along paths of lower viscosity gradients (Stehnach et al., 2021), analogous to Snell’s law of refraction (Gong, Shaik & Elfring, 2023), thereby minimizing viscous dissipation and motility costs. Is this strategy specific to swimmers, or do sinking species have their own strategies when facing high viscosity gradients? This is important to understand because it can influence the distribution of both swimmers and buoyancy-regulating microorganisms at different layers or depths within the ocean or lake community.

During vertical migration, species face turbulent flows of water. In response, sinkers and swimmers may employ different tactics such as chain formation (Fraga, Gallager & Anderson, 1989), morphology adjustments (Sengupta et al., 2017), or changes in orientation (Sengupta et al., 2017). This indicates that turbulence indirectly alters motility costs. How? Sudden events such as turbulence can trigger stress responses in organisms, leading to the production of reactive oxygen species, which in turn reduce the overall metabolic rate, making motility relatively more expensive. In addition, rapid bursts of movement in response to turbulence are energetically dissipative processes, and morphological adjustments can affect motility external efficiency.

The following three sections present the fundamental principles of swimming and buoyancy regulation in various phytoplankton species, which we consider essential groundwork before exploring the costs and benefits of motility. Section 2 explores key questions related to swimming: What are the different types of swimming machinery in phytoplankton? Where does the concept of swimming efficiency originate? Why is it important to understand flow velocity fields? And how do some swimmers achieve escape jumps? Section 3 examines buoyancy regulation: What mechanisms are used to control buoyancy? How are sinking and rising speeds estimated? What factors influence these vertical movements? And what are the dynamics behind sudden bursts of movements in some species? Section 4 introduces the environmental context: Why are phytoplankton exposed to extreme and fluctuating conditions? The answer lies in their migratory behavior, which subjects them to a wide range of light, nutrient, temperature, viscosity, turbulence, and biotic conditions (e.g., predators and viruses). Section 5 provides an analysis of both direct and indirect motility costs and benefits, including an example to illustrate how these costs can be quantified. Section 6 discusses how environmental parameters - particularly temperature, the main focus of this review - have multifaceted and often hidden effects on motility costs, complicating efforts to estimate them accurately. Section 7 explores whether intercorrelation between certain parameters - specifically organism size and speed - should be investigated as a potential source of motility cost.

## 2. Mechanisms and Dynamics of Phytoplankton Swimming

To estimate the operational costs of phytoplankton swimming, one must first understand the various mechanisms by which these organisms achieve motility. These mechanisms include: the use of flagella, as seen in dinoflagellates, chrysophytes, raphidophytes, haptophytes, and chlorophytes; the generation of surface waves powered by protein motors in some cyanobacteria; gliding via type IV pili in certain cyanobacteria; and specialized structures such as haptonema used for escape jumps. In addition to understanding the molecular basis of these motility machineries and their associated energetic demands, it is also essential to consider the efficiency of the swimming machinery involved. These two factors contribute to the operational cost of swimming. But how does swimming benefit the organism by generating feeding currents and enhancing nutrient uptake rates? To address this question, one must analyze the fluid velocity fields generated by flagella or other swimming mechanisms.

Most phytoplankton swimmers use flagella, which are long, whip-like structures common in eukaryotic cells that facilitate movement. These flagella typically consist of an internal structure called the axoneme, composed of a “9+2” arrangement of microtubules: nine outer doublets surrounding two central singlets (Schavemaker & Lynch, 2022). Protein complexes like dynein arms, nexin-DRC, and radial spokes link these microtubules, all enclosed within a membrane (Schavemaker & Lynch, 2022). Flagellate swimming speeds typically range from 10 to 1500 μm/s, with a median around 130 μm/s, based on data compiled by (Lisicki et al., 2019). The speed of flagellated phytoplankton and the flow fields they generate are influenced by the number of active flagella (Schavemaker & Lynch, 2022), flagellum length (Schavemaker & Lynch, 2022), beat frequency, beat amplitude, beat synchronization (Geyer et al., 2013), and flagellar arrangement (Nielsen & Kiørboe, 2021). Data on the number and length of flagella have been compiled for over 200 species of unicellular eukaryotes, including phytoplankton, and bacteria (Schavemaker & Lynch, 2022). As an example of the flagellar arrangement in phytoplankton species, dinoflagellates possess two flagella, one transversal and the other longitudinal (known as the trailing flagellum). Fenchel (Fenchel, 2001) observed that the trailing flagellum contributes to both the cell's translational velocity and its rotation around an axis that is perpendicular to its length. Meanwhile, the transversal flagellum mainly induces rotation around the cell's longitudinal axis.

There are alternative forms of swimming, other than employing flagella in phytoplankton, which are not easily observable. In *Synechococcus*, locomotion is facilitated by traveling waves along the surface of the cell body (Ehlers & Oster, 2012). These waves result from cyclic surface deformations driven by protein motors modeled as a rotating helix. Motors move faster than waves traveling on the body surface, and the waves move faster than the organism's swimming speed. The force required to propel the organism originates from the double integration of the stress tensor, which is influenced by the fluid velocity fields across the cell membrane surface (Ehlers & Oster, 2012). Consider a cell with a radius r of 1μm, a wave amplitude ϵr of 0.02 μm associated with waves on its surface, and a dimensionless wave number $k=\frac{2\pi r}{\lambda }$, where λ represents the wavelength. To achieve an organism velocity v of 15 μm/s, a wave speed $c=\frac{\omega r}{k}$ of 456 μm/s is necessary, where ω represents the angular frequency, as determined by the formula $v=82.2\epsilon^{2}c$ derived in (Ehlers & Oster, 2012). The fixed dimensionless coefficient 82.2 in this equation arises from an approximate representation of cell-surface deformations as Legendre polynomials and a fixed wave number k=16. For relatively large wave numbers, the result will change. For a complete discussion, see (Ehlers & Oster, 2012) and exploration of other models investigating the swimming velocity driven by surface-distorted waves, see (Ehlers et al., 1996; Stone & Samuel, 1996; Ehlers & Oster, 2012). The organism's speed contributes to the Stokes hydrodynamic swimming cost, while the wave speed on the surface of the cell body and the speed of motor proteins contribute to energy dissipation in the internal machinery.

The freshwater cyanobacterium, *Synechocystis* sp., exhibits a gliding or twitching-type motility with an average speed ranging from 0.09 to 0.15 μm/s (Samadi et al., 2023). This movement in water is facilitated by type IV pili (T4P), which are long, hair-like dynamic filaments on the organism's surface. The molecular structure of T4P is detailed in (Craig, Forest & Maier, 2019). Although the physics governing the velocity field patterns, thrust forces, and swimming efficiency of this cyanobacterium remain unexplored, it is suggested that its motility resembles that of soil bacteria such as *Myxococcus xanthus* (McBride, 2001). The motility of *Myxococcus xanthus*, viewed as an elastically flexible cell, is modeled by several forces: the forces generated by circular nodes (motor proteins) interconnected by angular springs, viscous forces, and interaction forces between nodes and substrates (Balagam et al., 2014). The retraction-elongation velocity of a single pilus fiber is approximately 1 μm/s with an average stall force of 110 pN (Maier et al., 2002), and this velocity is contingent upon the concentration of retraction motors (Clausen, Koomey & Maier, 2009). The stall force is the force at which the pilus fiber stops moving despite the continued application of force.

High-speed escape jumps, which are purposefully directed, have been observed in various phytoplankton swimmers, demonstrating their ability to evade predators by responding to fluid mechanical cues in the surrounding water (Jakobsen, 2002; Jiang et al., 2018; Miano et al., 2024). For instance, *Chrysochromulina simplex* has been shown to increase its swimming speed from approximately 1,400 μm/s to 6,600 μm/s (a nearly fivefold jump) in just 0.05 seconds (Jakobsen, 2002). However, jumping is not only a predator avoidance behavior. In the photosynthetic ciliate *Mesodinium rubrum*, spontaneous jumping serves as a key behavioral adaptation to overcome diffusion-limited nutrient uptake (Jiang & Johnson, 2017). The characteristics of these jumps vary with temperature, strain, and light conditions. Despite differing speeds and environments, both Antarctic and temperate strains of *M. rubrum* achieve similar jump distances (Jiang & Johnson, 2017). The Antarctic strain compensates for cold temperatures and reduced metabolic rates by performing longer-duration jumps with extended ciliary beat cycles, whereas the temperate strain also exhibits short-distance tumbling, which may further enhance nutrient uptake by generating local shear (Jiang & Johnson, 2017). The molecular mechanisms underlying escape jumps in microswimmers vary across species. In haptophytes such as *Chrysochromulina simplex*, the escape jump is a coordinated two-step process (Miano et al., 2024). First, the haptonema (a long, flexible appendage) rapidly coils, briefly pulling the cell forward. Immediately afterward, the two flagella reverse their beating direction and dramatically increase their speed, propelling the cell backward at high speed (Miano et al., 2024). The energetic cost of these high-speed jumps will be addressed in Section 5.

Phytoplankton swimmers operate in a low Reynolds number regime, where the dimensionless Reynolds number Re=rvρη is much less than one. In this regime, inertial forces are negligible compared to dominant viscous forces, fundamentally shaping how these organisms move. Here, r and v denote the organism’s characteristic size and swimming speed, while ρ and η represent the fluid’s density and dynamic viscosity, respectively.

The Navier-Stokes equation, which governs motility in a viscous fluid, describes the momentum balance in a Newtonian fluid based on Newton's second law. For Newtonian fluids, the viscosity remains constant, meaning the fluid’s resistance to flow does not change with the speed at which it flows or the amount of force applied. In the low Reynolds number regime, the inertial terms can be disregarded. Under these conditions, the drag force, F, acting on a spherical, rigid particle in laminar and uniform flow - assuming non-interfering particles - is given by F=6πηrv, where 6πηr is the drag coefficient and π≅3.14 is a constant. The minimum power required for swimming by a spherically shaped species is then:

(1). $$P=Fv=6\pi \eta rv^{2}$$

This equation has found extensive use in the literature for estimating the swimming cost of microswimmers (Raven & Richardson, 1984; Crawford, 1992; Katsu-Kimura et al., 2009; Beveridge et al., 2010). For a thorough derivation, readers are directed to fluid mechanics textbooks such as (Kundu, Cohen & Dowling, 2012), specifically the section titled 'Creeping Flow around a Sphere.' For detailed, biologically relevant discussions, see 'Foundations 16.2' in Ref. (Lynch, 2024). Note that the Equation (1) represents the minimum power needed, not the total power required for swimming. This distinction highlights the importance of the concept of swimming efficiency. Mechanical efficiency is defined as the ratio between the minimum power needed to tow an object (characterized by its drag coefficient at the swimmer’s average speed over a beating cycle), and the average power the swimmer transfers to the surrounding fluid during that same cycle (Lighthill, 1952; Guasto, Rusconi & Stocker, 2011). Several methods exist for determining the denominator of this ratio, though the underlying physics is quite complex and beyond the scope of this review. For instance, calculating the local velocities and corresponding forces along the length of the organism body and flagella (or other locomotion structures) typically involves applying theories such as resistive force theory or slender-body theory (Childress, 1981; Leal, 2007). These approaches require evaluating the strain rate tensor to capture the interactions with the surrounding fluid (Leal, 2007). Therefore, for the purposes of this review, we rely directly on empirical values of swimming efficiency reported in the literature to estimate the operational cost of swimming, as detailed in Section 5.

Looking at Equation (1), iťs important to note that viscosity is not necessarily constant in the ocean. Phytoplankton motility takes place within a viscosity gradient. Viscosity is influenced by water properties and by the biological activity of microorganisms within shear-induced aggregates or associated with extracellular polymeric substances (EPS) of high molecular weight, as well as light-induced lysis (Guadayol et al., 2021).

Recent microrheological techniques (Guadayol et al., 2021) have revealed that approximately 45% of the studied phytoplankton species (including cultures of diatoms, dinoflagellates, and haptophytes) experience viscosity levels up to 40 times that of artificial seawater within a 30 μm radius from the cell (phycosphere). According to (Guadayol et al., 2021), the relative viscosity is, on average, 2.6 times higher near the cell wall due to the production of extracellular polymeric substances, up to 30% higher near lysed and dead cells, and more than one order of magnitude higher within aggregates. How do these viscosity gradients affect the motility of phytoplankton, and do they employ any strategies in response? Microfluidic experiments involving *Chlamydomonas reinhardtii* by Stehnach et al. (Stehnach et al., 2021) indicate that these organisms encounter high local viscosities, which decrease their speed. Nevertheless, they exhibit adaptability by changing their strategy and reorienting down the gradient (Stehnach et al., 2021).

As a benefit of swimming, phytoplankton can use their motility apparatus to generate feeding currents in the surrounding fluid, thereby enhancing the likelihood of prey or particle capture and facilitating greater nutrient uptake. The resulting flow velocity fields - driven by forces associated with different flagellar configurations - determine the shape and extent of streamlines around the organism (Nielsen & Kiørboe, 2021) and can be modeled using idealized force-based approaches (see Table 1). These point-force models include the *stokeslet* (Pozrikidis, 1992), *puller stresslet* (Pozrikidis, 1992; Kiørboe et al., 2014), and *three-stokeslet quadrupole* (Andersen, Wadhwa & Kiørboe, 2015). They are formulated based on the action of one or more time-averaged point forces applied at reference points, with flow velocity fields derived from Green’s function solutions (Lauga & Powers, 2009). In these models, the propulsive force 𝐹 is considered proportional to the product of flagellum length, number of active flagella, beat frequency, and beat amplitude (Nielsen & Kiørboe, 2021). The modeled flow velocity fields can be used to estimate clearance rates - that is, the volume of fluid per unit time from which prey or particles can be captured. For quantitative estimates of clearance rates and numerical simulations of flow fields, the reader is referred to (Nielsen & Kiørboe, 2021). While this approach offers a mechanistic assessment of clearance rates generated by swimming, we solely rely on empirical equations in Section 5 to estimate the enhancement in nutrient uptake as a measure of motility benefit.

## 3. Essential Principles of Buoyancy Regulation

In this section, we discuss the Stokes-driven buoyancy equation, focusing on the parameters it captures completely, partially, or not at all. We explore the developed form of this equation, emphasizing the differences in mass density among various organelles of the species and their respective volumes, which can deviate from the mean mass density of the whole organism. The foundational knowledge presented in this section is essential for understanding and quantifying the cost of buoyancy regulation, encompassing both its molecular mechanisms and associated dissipative processes.

When a spherical rigid object falls through a fluid, three forces act upon it:

1. Gravitational Force: This force pulls the object downward and is determined by the objecťs mass, $F_{\text{gravity}}=mg$, where m is the object’s mass and g is the gravitational acceleration (standard value g=9.80665 m/s^2^). For a sphere, the mass is given by $m=\rho_{c}V$, where $\rho_{c}$ is the mass density of the sphere and $V=\frac{4}{3}\pi r^{3}$ is its volume. Substituting m, we get $F_{\text{gravity}}=\frac{4}{3}\pi r^{3}\rho_{c}g$.
2. Buoyant Force: This upward force arises due to the displaced fluid and is expressed as $F_{\text{buoyant}}=\rho_{w}Vg$, where $\rho_{w}$ is the mass density of the fluid.
3. Drag Force: This resistive force opposes the objecťs motion through the fluid. According to Stokes’ law, $F_{\text{drag}}=6\pi \eta rv$, where η is the dynamic viscosity of the fluid, r is the radius of the sphere, and v is its velocity. By balancing these forces, $F_{\text{gravity}}-F_{\text{buoyant}}=F_{\text{drag}}$. Substituting the expressions for each force and solving for v, the sinking/rising velocity is:
(2), $$v=\frac{2gr^{2}\left(\rho_{c}-\rho_{w}\right)}{9\eta }$$

where $\rho_{c}-\rho_{w}$ represents the difference between the mass density of the object (e.g., a sinking or rising phytoplankton) and the fluid (e.g., seawater or freshwater). While treating sinking phytoplankton as passive particles implies that sinking, regardless of speed, incurs no direct energetic cost to the organism (because organism does not pay for the dissipation - gravity does the work), this perspective overlooks the reality that many species actively regulate their buoyancy (and thus their sinking or rising speed). When viewed as active organisms, their motility costs can be meaningfully estimated based on the metabolic energy required for buoyancy control mechanisms, which, based on Equation (2), depend at least on density adjustments and shape adaptations. In this section, we discuss the factors that actively influence sinking and rising speeds, which directly impact the associated energetic costs.

In general, intracellular macromolecules like proteins and carbohydrates typically possess a higher mass density (~ 1300 and 1500 kg.m^−3^ respectively) than seawater (~ 1030 kg.m^−3^), whereas lipids have a lower mass density (~ 860 kg.m^−3^) in comparison to water (Boyd & Gradmann, 2002). Phytoplankton exhibit varying density differentials relative to their environment. This density differential causes vertical migration, both upward and downward, over the course of a day, several days, or even up to a week (Wirtz & Smith, 2020). Phytoplankton employ several mechanisms to adjust their internal density. Eukaryotic phytoplankton may replace lighter ions with heavier ones, or vice versa (Raven & Lavoie, 2021), accumulate ballast materials (Raven & Lavoie, 2021), or incorporate ammonium derivatives such as trimethyl ammonium (~ 993 kg.m^−3^) (Boyd & Gradmann, 2002) into their vacuoles to adjust their sinking or rising behavior. In contrast, cyanobacteria manipulate gas vesicles (Raven & Lavoie, 2021) to regulate buoyancy. The mass density of phytoplankton typically ranges from 920 to 1085 kg/m^3^ for cyanobacteria, 1020 to 1140 kg/m^3^ for Chlorophyta, and 1009 to 1263 kg/m^3^ for Bacillariophyta (Moore & Villareal, 1996a; Reynolds, 2006; Woods & Villareal, 2008).

Density variations in diatoms arise from a complex interplay among factors such as the heavy siliceous frustule (denser than 2000 kg.m^−3^), ammonium derivatives, cell shape, nutrient flux, silica requirements, and large vacuoles which are influenced by environmental conditions and grazing pressure (Boyd & Gradmann, 2002; Finkel & Kotrc, 2010). The silicification process, which defines the frustule, significantly influences vertical migration of diatoms when high energy resources are not available (Raven & Waite, 2004; Petrucciani et al., 2023). When faced with nutrient limitations and iron deficiencies, diatoms tend to exhibit higher Si:C ratios, promoting faster sinking rates to access nutrient-rich deep ocean layers (Finkel & Kotrc, 2010).

Are diatoms limited to sinking, or can they regulate their buoyancy to become neutral or positively buoyant? Numerous studies have demonstrated that diatoms are capable of achieving neutral or even positive buoyancy. For instance, laboratory experiments reveal that *Thalassiosira weissflogii* maintains neutral buoyancy under nutrient-rich conditions but begins to sink when nutrients are scarce (Richardson & Cullen, 1995). Similarly, in *Rhizosolenia formosa H. Peragallo*, nitrate depletion reduced the proportion of positively buoyant cells from 11% to 4%, which rebounded to 9% within 12 hours of nitrate resupply (Richardson et al., 1996). Research on the genus *Rhizosolenia* also indicates that buoyancy direction is influenced by light intensity, likely through a mechanism involving carbohydrate ballasting (Moore & Villareal, 1996b). Field studies further support this view: prior to the spring bloom, non-growing diatoms tend to sink, whereas during the bloom, they exhibit neutral buoyancy, suggesting that buoyancy regulation is closely linked to their growth status (Acuña et al., 2010).

Large diatoms like *Nitzschia palea* can sink rapidly, reaching speeds of up to 43 m/d or 500 μm/s (Durante et al., 2019). These speeds might be variable, exhibiting an unsteady sinking pattern observed in large species like *Coscinodiscus wailesii* (Gemmell et al., 2016; Du Clos, Karp-Boss & Gemmell, 2021). This behavior involves bursts of rapid sinking with a 1.5% change in mass density, coupled with periods of slow or near-zero speeds. Such fluctuating behavior among diatoms over timescales of seconds seems to confer a competitive advantage in nutrient assimilation (Gemmell et al., 2016). Nevertheless, this fluctuating behavior appears absent under dark conditions, at least in the case of *Coscinodiscus wailesii* (Du Clos et al., 2019).

It is traditionally believed that lipids, despite having a lower mass density than water, do not play a major role in buoyancy regulation (Boyd & Gradmann, 2002). This is primarily because vacuoles (Boyd & Gradmann, 2002; Behrenfeld et al., 2021), which occupy a considerable portion of the cell volume (typically 30% in relatively small diatoms to 90% in relatively large diatoms (Raven, 1995)), and silica-rich heavy frustules play the major role in buoyancy regulation. However, it has been reported that there is a strong positive correlation between the increased sinking speed of *Phaeodactylum tricornutum* diatoms (nearly threefold) and the rise in their lipid content under phosphate limitation or depletion (Zhang et al., 2023). This may suggest that changes in lipid composition lead to structural alterations, such as modifications in membrane rigidity, which result in changes in cell shape and size (Harayama & Riezman, 2018; Lee et al., 2024), and/or changes in the interactions between lipid droplets and vacuoles (Olzmann & Carvalho, 2019; Tanaka et al., 2024). All of these factors could indirectly influence sinking behavior.

Theoretical models have been developed to predict the maximum sinking rate of cylindrical and spherical diatoms with greater precision than conventional Stokes law (Miklasz & Denny, 2010). Based on the model in (Miklasz & Denny, 2010) the sinking speed of spherical diatoms is given by:

(3), $$v=\frac{2g}{9\eta }\left[\rho_{\text{cyt}}\frac{{\left(r-t\right)}^{3}}{r}+\rho_{\text{fr}}\frac{\left(3r^{2}t-3rt^{2}+t^{3}\right)}{r}-\rho_{\text{w}}r^{2}\right]$$

where $\rho_{\text{cyt}}$, $\rho_{\text{fr}}$, and $\rho_{\text{w}}$ denote the mass densities of the cytoplasm, frustule, and seawater, respectively, and $t=b_{1}r^{a_{1}}$ represents the radius-dependent frustule thickness (with dimensions of length). Here, $a_{1}$ and $b_{1}$ are scaling coefficients, and each term in the brackets has the dimension of mass per unit length (ML^−1^). The cell density is $\rho_{\text{cell}}=\rho_{\text{cyt}}\frac{V_{\text{cyt}}}{V_{\text{cell}}}+\rho_{\text{fr}}\frac{V_{\text{fr}}}{V_{\text{cell}}}$, where $V_{\text{cyt}}$, $V_{\text{fr}}$, and $V_{\text{cell}}$ represent the volumes of the cytoplasm, the frustule, and the cell. One suggestion for future research is to incorporate the significant influence of vacuole mass density ($\rho_{\text{vac}}=1017.6$ kg.m^−3^ (Raven & Doblin, 2014)), due to their considerable volume and role in positive buoyancy (RAVEN, 1987, 1995; Raven & Lavoie, 2021), into Equation (3). This factor reduces overall cell density, thereby facilitating positive buoyancy (Lavoie & Raven, 2020). The approach to do this is to add a new term in the form of $\rho_{\text{vac}}\frac{V_{\text{vac}}}{V_{\text{cell}}}$ into Equation (3). To determine the volume of the vacuole relative to the cell volume, data provided in (Raven, 1995) for diatoms can be used. By fitting a function to these data, the ratio $\frac{V_{\text{vac}}}{V_{\text{tot}}}$ for different cell volumes can be obtained. For example, in a cell with a volume of 5.4×10^4^ μm^3^, approximately 90% of the protoplast volume is occupied by the vacuole (Raven, 1995), making the vacuole-to-cell volume ratio significantly higher than the relative contributions of the frustule and the remaining cytoplasm.

How much variation in density is possible, and how long does it take for the buoyant cell to achieve this? According to (Larson et al., 2024), *Pyrocystis noctiluca,* a non-flagellated dinoflagellate, experiences a six-fold increase in cellular volume within a span of less than 10 minutes. This process involves the calcium-activated influx of *fresh* water through aquaporin channels, followed by transport to the vacuole, resulting in the reversal of gravitational sedimentation (Larson et al., 2024). This creates a situation where the cell mass density, $\rho_{\text{cell}}=\rho_{\text{cyt}}\frac{V_{\text{cyt}}}{V_{\text{cell}}}+\rho_{\text{vac}}\frac{V_{\text{vac}}}{V_{\text{cell}}}$, becomes less than the mass density of seawater.

The sinking and rising dynamics expressed in the form of Equations (2) and (3) do not incorporate the shape factor as a parameter. Non-spherical geometric shapes generally sink at a slower rate compared to spheres of the same volume (Padisák et al., 2003; Naselli-Flores & Barone, 2011). However, tear-drop-shaped phytoplankton (e.g. *Rhodomonas* and *Gymnodinium*) serve as an exception, sinking faster than their spherical counterparts (Padisák et al., 2003; Naselli-Flores & Barone, 2011). To the best of our knowledge, there is no clear physical or physiological principle that explains the functional efficiency of different shapes or how shape influences the energetic cost of buoyancy regulation.

## 4. Phytoplankton Migration: Survival Strategies

Why is estimating the cost of motility a time-dependent, dynamic process? Because phytoplankton engage in migratory behaviors that expose them to constantly changing environmental conditions, which directly and indirectly influence their motility costs and benefits. These varying conditions include changes in temperature, light, nutrient availability, viscosity, predation and viral risks, and turbulence. Therefore, before examining the costs of migration, it is essential to understand the fundamentals of phytoplankton migration. In this section, we discuss the time-scale and length-scale of vertical migration for both relatively slow and fast phytoplankton, and their role in net primary production. We examine the velocity at which migrating phytoplankton reach their target depth, the impact of increasing speed on the accumulation of species at specific depths, and the influence of turbulence on diversity. Additionally, we explore the strategies phytoplankton employ in turbulent waters, such as chain formation, division into sub-populations, and changes in orientation, direction, and shape.

Phytoplankton primarily migrate by orienting themselves through chemotaxis, phototaxis, and gravitaxis - movements in response to chemical gradients, light, and gravity, respectively. When species migrate either towards the ocean's surface, where light intensity is higher, or to deeper depths with greater nutrient concentrations, there exists the potential for an increase in their rates of photosynthesis or nutrient uptake (Raven & Richardson, 1984; Ji & Franks, 2007). The light-rich and nutrient-rich regions are typically separated by distances of 30–120 m, and relatively fast phytoplankton species are capable of vertical diel migration within these regions (Raven & Richardson, 1984; Ji & Franks, 2007). Organisms that migrate diurnally to the surface during the light period may form a subsurface layer when faced with nutrient limitations (e.g., nitrate), where light and nutrient availability are optimally balanced (Cullen & Horrigan, 1981). For a comprehensive review of the mechanisms underlying phytoplankton layer formation - including straining, convergent swimming, buoyancy regulation, gyrotactic trapping, *in situ* growth, and intrusions - see (Durham & Stocker, 2012).

Phytoplankton species use different depth-regulation strategies - mixing, migration, and layer formation - based on their motility and adaptation to specific hydrographic regimes (Cullen & MacIntyre, 1998). These strategies involve ecological trade-offs in physiology and behavior, including photoacclimation, motility control, nutrient storage and uptake, and photoprotection (Cullen & MacIntyre, 1998). Together, these traits define species-specific ecological niches and help explain the success of certain phytoplankton in forming harmful algal blooms under varying oceanographic conditions.

During vertical migration, the species may also exhibit limited horizontal movement, relative to vertical migration, through diffusion alone, typically spanning up to 10 centimeters, as observed in ideal lab experiments in the absence of water currents (Schuech & Menden‐Deuer, 2014). This horizontal displacement is relatively insignificant compared to their predominant vertical movement; however, tidal currents can significantly influence the horizontal movement of species (Sala et al., 2023).

Contrary to the common assumption that most phytoplankton species lack migratory behaviors due to their relatively slow speed, Wirtz and Smith (Wirtz & Smith, 2020; Wirtz et al., 2022) reported that the observed depth-dependent profile of chlorophyll-a concentration (Cullen, 2015) in the oligotrophic ocean is attributed to long-term (days to week) slow vertical migration via sinking/rising or swimming processes. This slow form of active mobility, which is considerably more challenging to track compared to diel migration, contributes to 7–28% of the ocean's net primary production (Wirtz & Smith, 2020; Wirtz et al., 2022). The variation in the nutrient-to-carbon ratio at identical ocean depths is suggested to result from distinct migration histories, involving movement either from the upper surface ocean to deeper depths or vice versa (Wirtz et al., 2022).

Klausmeier and Litchman (Klausmeier & Litchman, 2001) developed a dynamic mass-balance model that considers swimming/sinking speed as a parameter, integrating factors such as growth rate under light and nutrient limitation, loss rate, eddy diffusion, and active movement to predict the vertical distribution of phytoplankton in poorly mixed water columns. Active movement is expressed as a term in a time-dependent biomass-balance equation, represented as a one-dimensional gradient of the product of speed, biomass $\left(B\right)$, and the gradient of the growth rate, written as $\frac{\partial }{\partial z}\left(v\frac{\partial \mu }{\partial z}B\right)$. Their findings demonstrate that as phytoplankton speed increases, they tend to concentrate within a narrow layer at a specific depth, where they experience an equal limitation of light and nutrients. This depth represents an evolutionarily stable strategy (Klausmeier & Litchman, 2001).

Turbulence is an inherent characteristic of oceanic waters that significantly affects phytoplankton, impacting their mortality rate, motility costs, distribution, and diversity (Lévy et al., 2014). As a result, phytoplankton must develop strategies to adapt to turbulent conditions. In typical estimations, iťs often assumed that the speed of both vertical and horizontal water currents is insignificant compared to that of microswimmers/sinkers, simplifying calculations. However, when confronted with relatively faster water currents, such as rip currents, eddies, and turbulence, microswimmers and sinkers employ strategies that have been observed in lab experiments. The fundamental physics governing some idealized strategies of a model swimmer, such as swimming towards safer points, minimizing swimming time and path in parallel currents, source currents, and sink currents, is explored in (Aghamohammadi, Aghamohammadi & Moghimi-Araghi, 2023).

Migrating phytoplankton demonstrate the ability to re-orient their swimming trajectory in response to turbulence (Smayda, 2010) or engage in collective behavior by forming elongated chains, as observed in diatoms, cyanobacteria, and dinoflagellates within the water column (Sullivan et al., 2003; Smayda, 2010; Lovecchio et al., 2019). The formation of chains enhances swimming/sinking speeds particularly in weak to moderately turbulent water flows (Lovecchio et al., 2019). This occurs because as the number of cells in a chain increases, the collective propulsive force outweighs the hydrodynamic drag acting on the chain (Fraga et al., 1989). The swimming speed of the chain can be determined by considering both the number of cells comprising the chain and the chain's shape factor (Lovecchio et al., 2019) as following:

(4), $$v_{\text{c}}^{\left(n\right)}=n^{\left(\frac{2}{3}\right)}\frac{v_{\text{c}}^{\left(1\right)}}{K}$$

where $v_{\text{c}}^{\left(n\right)}$, n, $v_{\text{c}}^{\left(1\right)}$, and K represent, respectively, the chain swimming speed, the count of rigidly attached spherical cells, the swimming speed of a single species, and the shape correction factor. While Equation (4) describes how swimming speed scales with chain length and geometry, estimating the metabolic cost of swimming requires applying Stokes' law in conjunction with an assessment of the chain’s mechanical efficiency - a key factor influencing total energy expenditure that, to the best of our knowledge, remains unexplored.

In turbulent hydrodynamic conditions, it has been reported that dinoflagellates and raphidophytes may divide into two subpopulations within approximately 30 minutes (Sengupta et al., 2017). One group propels upwards while the other navigates downwards, facilitated by geometric shape adjustments to their motility mechanisms (Sengupta et al., 2017) which directly affect motility efficiency. To model the dynamic morphological changes, particularly the fore-aft asymmetry discussed in (Sengupta et al., 2017), it is necessary to investigate the center of buoyancy, center of mass, center of hydrodynamic stress, and the organism's orientational stability. While such rapid responses to severe environmental shifts may expose the microorganism to elevated cellular stress (characterized by nitric oxide (NO) production) and incur significant costs, they underscore the species' advanced survival strategies.

## 5. Trade-offs of Phytoplankton Movement: A Cost-Benefit Perspective

Swimming and buoyancy regulation have metabolic costs for both the costruction (e.g., biosynthesis of flagella) and operation. We can analyze our understanding of the factors influencing migratory behavior by analyzing these costs and comparing them to the benefits of motility.

### 5.1. Benefit Analysis

Motility enables phytoplankton to reorient along the light vector through phototaxis (Jékely, 2009), enhancing photosynthetic efficiency, and to navigate toward higher nutrient concentrations via chemotaxis (Blackburn & Fenchel, 1999; Govorunova & Sineshchekov, 2005). Both processes rely on complex signal transduction pathways (Lynch, 2024). Additionally, as discussed in Section 2, the motility apparatus itself (such as specific flagellar arrangements) (Nielsen & Kiørboe, 2021) can generate feeding currents that improve nutrient access. Jumping-like movements also contribute significantly to overcoming diffusion limitations and enhancing nutrient uptake (Jiang & Johnson, 2017).

The Sherwood number (Sh) is a dimensionless number that quantifies how much faster phytoplankton can take up nutrients by moving compared to relying solely on passive diffusion. This enhancement of nutrient uptake during swimming or sinking/rising results from advection and is expressed as (Langlois et al., 2009; Nielsen & Kiørboe, 2015):

(5), $$\text{Sh}=\frac{Q}{4\pi rDC_{\infty }}$$

in which Q, r, D, and $C_{\infty }$ represent, respectively, the nutrient flux, cell radius, diffusion coefficient, and nutrient concentration at a distance far from the cell. A stationary cell is characterized by an Sh of 1 while an Sh of 1.2 signifies a 20% enhancement in nutrient uptake rate attributed to swimming/sinking. For example, empirical investigations of the flow field around the dinoflagellate *Dinophysis acuta* (79 μm in length, 54 μm in width, 104 μm/s swimming speed, $D=10^{-9}\;{\text{m}}^{2}/\text{s}$, with constant nutrient concentration far from the cell and zero nutrient concentration at the cell surface) show that swimming contributes to a substantial 75% increase (Sh = 1.75) in nutrient uptake rate compared to diffusion alone (Nielsen & Kiørboe, 2015). Jumping behavior in photosynthetic *Mesodinium rubrum* significantly enhances nutrient uptake by increasing the Sherwood number (Jiang & Johnson, 2017). A one-beat short jump in Antarctic strains can raise Sh to 2.5, while multiple-beat long jumps reach up to 3.3. In temperate strains, long jumps peak at Sh = 3.9, indicating more efficient nutrient transport. Additionally, brief episodes of fast sinking are reported to allow *Coscinodiscus wailesii* diatoms to enhance nutrient uptake by 170% compared to when they sink at their usual rate (Gemmell et al., 2016).

To calculate the Sherwood number, one can employ the correlation between the Sherwood number and the Peclet number, $\text{Pe}=\frac{rv}{D}$, developed for a spherical steady swimmer (Magar, Goto & Pedley, 2003), a monoflagellated organism (Langlois et al., 2009), and a biflagellated organism (Tam & Hosoi, 2011). An example of such an empirical relationship for a sinking sphere found in the literature is (Clift & Grace, 1978):

(6). $$\text{Sh}=\frac{1}{2}\left(1+{\left(1+2\text{Pe}\right)}^{1/3}\right).$$

Given that the speed and characteristic radius of phytoplankton species are documented in the literature, it is straightforward to estimate the Sherwood number and consequently evaluate the enhancement in nutrient uptake attributed to swimming or sinking using Equation (6). For example, for the spherical-shaped sinking coccolithophore *Calcidiscus leptoporus* with a radius of approximately 10 μm and a mean sinking rate of 4.3 m/d (Durante et al., 2019), the Peclet number is calculated as: $\text{Pe}=\frac{10\times 10^{-6}[\text{m}]\times 4.3\times {\left(86400\right)}^{-1}\left[\frac{\text{m}}{\text{s}}\right]}{10^{-9}\left[\frac{{\text{m}}^{2}}{\text{s}}\right]}≅0.5$. This gives the Sherwood number as $\text{Sh}=\frac{1}{2}\left(1+{\left(1+2\times 0.5\right)}^{1/3}\right)≅1.13$ indicating a 13% increase in the nutrient uptake rate.

As an approximation, it can be shown that under nutrient-limited conditions, the ratio of the Sherwood number to the dimensionless relative flagellar cost $C_{\text{rel}.}$ (which includes both construction and operating costs) yields the relative growth rate $\mu_{\text{rel}.}$ - that is, the growth rate of a motile cell relative to a stationary one (Schavemaker & Lynch, 2022):

(7). $$\mu_{\text{rel}.}=\frac{\text{Sh}}{1+C_{\text{rel}.}}.$$

According to the estimate outlined in Equation (7), eukaryotic flagellated microswimmers with a volume exceeding 1000 μm^3^ encounter an increase in their growth rate, while those with a volume less than 100 μm^3^ find nutrient gathering by flagella to be uneconomical, since its cost outweighs its benefits (Schavemaker & Lynch, 2022). This suggests that there might be other benefits to employing flagella in relatively small species, besides enhancing nutrient uptake rate.

### 5.2. Cost Analysis

Motility costs consist of any costs associated with motility from the time the organism starts moving due to a motive until it stops. These include: 1) the cost of constructing motility-related machinery, such as visible structures like flagella or non-visible components found in cyanobacteria, diatoms, etc.; 2) the minimum power required for swimming (determined by the drag force exerted on the organism due to viscosity and pressure) and for buoyancy regulation (based on molecular mechanisms of shape and mass density adjustments); 3) the cost related to the efficiency of motility-related internal and external machinery and external organism shape; and 4) the costs associated with any environmental changes during migration. Some costs occur at specific points during the cell cycle - such as the synthesis, remodeling, and degradation of motility machinery - and are considered one-time construction expenses, typically measured in joules or ATP per cell. In contrast, other costs are incurred continuously over time (operational costs) and are measured in joules or ATP per second per cell. The energy released from hydrolyzing one mole of ATP ranges from 28 kJ/mol under standard conditions to approximately 55 kJ/mol in *E. coli* during aerobic exponential growth, and can reach up to 72 kJ/mol in *Homo sapiens* muscle during intense exercise. This variation depends primarily on intracellular pH and magnesium ion concentration, as Mg^2+^ stabilizes ATP by binding to its phosphate groups (Milo & Phillips, 2015). For all calculations in this study, we adopt a representative value of 50 kJ/mol.

#### 5.2.1. Construction Cost of Flagellum

One notable expense associated with the phytoplankton swimming process is the construction cost of the flagellum. While it might be assumed that this is a one-time occurrence and therefore not a substantial energy fraction over the lifespan, research indicates that microorganisms can lose their flagella either accidentally or through programmed mechanisms. For instance, bacteria have the ability to remove or deactivate their flagellum in conditions of nutrient starvation (Ferreira et al., 2019; Zhu & Gao, 2020). Consequently, it is important to account for a flagellum loss rate when studying phytoplankton swimmers.

The construction cost refers to the expenses associated with building the proteins and lipids necessary for the formation of a flagellum. This is calculated by considering the number of proteins, the length of the proteins in terms of amino acids, and the average energy cost per amino acid as proposed by (Schavemaker & Lynch, 2022). Additionally, because eukaryotic flagella are enclosed by a membrane, the construction cost of the membrane should also be included. According to the estimation provided by Schavemaker and Lynch, without accounting for flagella loss rate, the costs of constructing flagella relative to the cost of constructing the whole cell in eukaryotes, assuming a spheroidal cell shape, are suggested to range between 0.037% and 44%, with a median value of 3% (Schavemaker & Lynch, 2022). The absolute cost of constructing flagella rises with cell volume because the total flagella length (product of the number and length of flagella) increases with cell volume, albeit with varying exponents among different groups. However, relative to the total construction cost of the cell, the cost of flagella construction tends to decrease as cell volume increases (Schavemaker & Lynch, 2022).

The cost associated with any modification to the flagellum, such as adding a single amino acid to the protein's structure, varies depending on the type of protein involved. This range extends from the relatively low cost of inner-arm dyneins to the higher cost of tubulins, in relation to the overall cell construction cost (Schavemaker & Lynch, 2022). The cost assessment of modifications in the flagella is important because the length of flagella varies within-cell due to biological fluctuations connected with LF genes (specifically LF1 (Nguyen, Tam & Lefebvre, 2005; Bauer et al., 2021)), which regulate the flagellum length-control machinery and lead to the addition or removal of a large number of tubulins (Bauer et al., 2021). The system regulating flagellar length in *Chlamydomonas reinhardtii* displays fluctuations significantly greater than measurement error, with a variability coefficient ranging from 10% to 20% (Bauer et al., 2021). Therefore, the cost associated with these within-cell flagella length fluctuations (Bauer et al., 2021), which involve the removal and addition of amino acids from/to proteins constructing the flagella, is important, as estimated in (Schavemaker & Lynch, 2022). However, there should always be a break-even point where further increases or decreases in flagella length become ineffective for the cell. The length should be adjusted to maintain a balance between costs and benefits, ultimately leading the cell to adopt an optimal intermediate length.

A microswimmer with a higher construction cost of flagella (due to having more and/or larger flagella) possesses the capability to achieve greater swimming speeds, as the log-log experimental data shows (albeit with a low R^2^ ~ 0.35) (Schavemaker & Lynch, 2022), leading to increased drag force and power consumption in a viscous environment.

#### 5.2.2. Construction Cost of Non-visible Swimming/Sinking Machineries

Cyanobacteria such as *Synechococcus* and *Synechocystis* sp. swim using non-visible machinery including protein motors that generate traveling waves on the cell surface, but little is known about the construction cost of the components involved in the propulsion of these organisms. Here, we provide a biological overview of the construction cost of these non-visible motility apparatuses. However, precise estimation of the associated costs remains a subject for future studies.

The essential components for the swimming of *Synechococcus* include the crystalline S-layer (surface layer) (Samuel, Petersen & Reese, 2001) composed of elongated subunits tilted approximately 60° with respect to the cell wall, SwmA glycoproteins (Brahamsha, 1996), SwmB polypeptides (McCarren & Brahamsha, 2007), and protein motors associated with the peptidoglycan layer (Ehlers & Oster, 2012). To estimate the construction cost of these motility-related components, one needs to determine the number and length of the subunits of the S-layer, SwmA, SwmB, and motor proteins. To the best of our knowledge, the copy number of all these components is not available in the literature.

The construction cost of motility-coupled components in *Synechocystis* sp. is linked to the copy number of type IV pilus (T4P) machines. These machines are composed of major and minor pilins, secretin, alignment proteins, platform proteins, assembly ATPase, and retraction ATPase (Craig et al., 2019). To estimate the construction cost of the T4P in *Synechocystis* sp., we refer to the PDB ID 3JC8, which represents the "Architectural model of the type IVa pilus machine in a piliated state" of *Myxococcus xanthus* (Chang et al., 2016). This structure, determined using electron microscopy, consists of 37,468 amino acid residues (Chang et al., 2016). Recent measurements indicate that the average copy number of T4P machines in *Synechocystis* sp. varies between 15 and 20 per cell (Chandra, Joubert & Bhaya, 2017). The final piece needed to calculate the total construction cost is the ATP hydrolysis cost per amino acid, which is provided below.

Constructing a single amino acid under aerobic conditions with glucose as the primary carbon source involves both a direct ATP cost and an opportunity cost. The opportunity cost refers to the energy the cell forgoes by diverting a metabolic intermediate into biosynthesis rather than fully oxidizing it for energy. For example, synthesizing alanine from pyruvate requires a direct expenditure of 2.5 ATP equivalents. However, if the cell had instead continued metabolizing pyruvate through energy-generating pathways, it could have yielded an additional 12.5 ATP equivalents. Therefore, the total cost of producing alanine is the sum of these two values: 15 ATP equivalents. Detailed breakdowns of the direct, opportunity, and total ATP costs for all 20 amino acids are provided in Foundations 17.2 of (Lynch, 2024). We downloaded the FASTA sequence file for PDB ID 3JC8 to count the total number of each amino acid type. By multiplying these counts by the ATP hydrolysis cost per amino acid type (as provided on page 435 of (Lynch, 2024)), we obtain an average (total) construction cost of 26.3 ATP hydrolysis events per amino acid for this specific T4P machine. Using this value, the total construction cost of the T4P machines can be estimated as: 20 [machines] × 37,468 [residues per machine] × 26.3 [ATPs per residue] = 19,708,168 ATPs, or approximately 2.0 × 10^7^ ATP hydrolysis events.

Given the potential differences in the number of residues between T4P of *Myxococcus xanthus* and *Synechocystis* sp., we also propose an alternative approach. We use the reported size of T4P machines in *Synechocystis* sp. (Chandra et al., 2017) to estimate its volume. Using the average volume per amino acid residue (1.33 × 10^−10^ μm^3^ (Schavemaker & Lynch, 2022)), we can then determine the number of residues per T4P machine. The average length and width of T4P machines in *Synechocystis* sp. are reported to be 2.5 μm and 0.007 μm, respectively (Chandra et al., 2017). Assuming the T4P structure is cylindrical, this gives an approximate volume of 9.6 × 10^−5^ μm^3^. Dividing this volume by the average volume per amino acid residue (assuming a packing fraction of 100%, i.e., that amino acids completely fill the volume of the cylindrical T4P structure) yields approximately 723,393 residues per T4P machine in *Synechocystis* sp., which is about 20 times higher than the residue count derived from the PDB ID of the *M. xanthus* T4P. Therefore, the total construction cost of the T4P machines in *Synechocystis* sp. can be calculated as: 20 [machines] × 7.2×10^5^ [residues per machine] × 26.3 [ATPs per residue] = 3.8×10^8^ ATP hydrolysis events. Assuming that T4P machines are the sole contributors to motility in *Synechocystis* sp., our estimated construction cost aligns with the median construction cost of all flagella of bacteria as reported in (Schavemaker & Lynch, 2022).

The construction cost associated with buoyancy regulation in phytoplankton varies with the number and size of proteins associated with the synthesis of gas vesicles in cyanobacteria (Walsby, 1994), the need to syntheses organic osmolytes (Raven & Doblin, 2014), the construction cost of pores and channels that mediate the active influx of water from the cytosol into the vacuole (Raven & Doblin, 2014), and the replacement of heavy ions with light ions in the vacuole (Raven & Waite, 2004; Raven & Doblin, 2014). In diatoms, the energy investment in frustule formation, which is strongly related to sinking, entails silica precipitation, cytoskeletal organization of silica-rich vesicles, silica metabolism regulated by genes, and assembly costs (Raven & Waite, 2004; Finkel & Kotrc, 2010). The calcification and formation of the coccolith layer in coccolithophores play a similar role in contributing to the sinking patterns of these microorganisms (Monteiro et al., 2016). One challenge in determining the construction cost of the components regulating phytoplankton buoyancy is that these components serve multiple purposes, making it difficult to estimate the fraction of energy expenditure specifically related to buoyancy regulation. For gas vesicles to provide buoyancy, 3 to 10 percent of the cell volume should be occupied by gas vesicles, depending on their mass density (Walsby, 1994). Approximately 10% of this volume is composed of the protein wall, which has a mass density of around 130 kg/m^3^, while the remaining volume is gas space, with a mass density of about 1 kg/m^3^ (Walsby, 1994). According to estimates by Walsby (Walsby, 1994), the construction cost of gas vesicles in *Escherichia coli* to achieve neutral buoyancy is approximately 2.84×10⁻^10^ J, which is roughly equivalent to the energy content of 3.42×10^9^ ATP molecules.

#### 5.2.3. Swimming External and Internal Efficiency

Mechanical efficiency refers to how effectively a swimmer converts its energy into motion, quantified as the ratio of the minimum power required to pull the organism - based on its drag at the average swimming speed over one beating cycle - to the average amount of power it actually delivers to the fluid during that cycle (Lighthill, 1952; Guasto et al., 2011). The swimming efficiency of flagellated unicellular organisms, whether eukaryotes or bacteria, is generally below 3% (Purcell, 1977, 1997; Stone & Samuel, 1996; Chattopadhyay et al., 2006; Guasto, Rusconi & Stocker, 2011; Tam & Hosoi, 2011; Arroyo et al., 2012; Barsanti et al., 2016). For example, a low efficiency of 1% means that the actual power required for swimming is 100 times greater than the minimum estimated using Stokes' law (Equation 1). This energetic cost can be substantial relative to the organism’s total metabolic rate, especially under nutrient-limited conditions.

Swimming efficiency can be divided into external and internal efficiency. External efficiency encompasses energy dissipation related to the swimmer's external characteristics, such as body geometric shape (Nasouri et al., 2021), flagellum-to-body length ratio (Tam & Hosoi, 2011; Barsanti et al., 2016), and flagellum slenderness (Tam & Hosoi, 2011; Barsanti et al., 2016), all of which are affected by the hydrodynamic viscosity of the environment. In contrast, internal efficiency pertains to energy dissipation within the organism, such as the elastic bending of the axoneme (Spagnolie & Lauga, 2010; Chen et al., 2015). The external efficiency associated with the physical properties of flagella has been reported to be 0.8% for the green alga *Chlamydomonas* (Tam & Hosoi, 2011) and 1.4% for *Tetraflagellochloris mauritanica* (Barsanti et al., 2016).

As described above, the external efficiency depends, in part, on the shape of the microswimmer. Ignoring the trade-off between microswimmer shape, navigation cost, and optimal navigation time (Piro et al., 2024), higher external efficiency values are observed for prolate-shaped microswimmers. These values decrease as the shape transitions towards more compact forms (such as spherical or cubic), with the lowest values found in oblate shapes (Vilfan, 2012; Guo et al., 2021; Nasouri et al., 2021). The maximum external efficiency or minimum dissipation for various geometric shapes is determined by the following formula. In Equation (8), $R_{\text{PS}}$ represents the drag coefficient of a perfect-slip rigid body, while $R_{\text{NS}}$ denotes the drag coefficient of a no-slip rigid body (Nasouri et al., 2021):

(8). $$\xi_{m}\leq 1-\frac{R_{\text{PS}}}{R_{\text{NS}}}$$

The result of Equation (8) is shown in Figure 3 of (Nasouri et al., 2021). To simplify the estimation of shape-related efficiency, a sigmoid function can be fitted to that figure, allowing efficiency to be estimated using only the shape's aspect ratio. This approach is applied as a working example in Section 5.3.

The theoretical model of (Giri & Shukla, 2022) shows that, for microswimmers using tangential surface actuation - where the surface slides fluid backward, like surface treadmilling, to move forward - the power required for swimming can vary by more than six orders of magnitude depending on the shape’s aspect ratio. We integrate the theory developed by these authors (Giri & Shukla, 2022) with empirical data on the elongation and aspect ratio of phytoplankton species (Ryabov et al., 2021). For example, we find that the external energy dissipation during swimming in certain *Ceratium* species, a genus of dinoflagellates characterized by an aspect ratio of 0.025 and oblate elongation, is exceptionally higher compared to certain *Closterium* species, a genus of Charophyte green algae characterized by an aspect ratio of 112.83 and prolate elongation, differing roughly by six orders of magnitude.

The examination of over 5700 phytoplankton species' shapes spanning 402 genera reveals they can be categorized into 38 distinct geometric forms (Ryabov et al., 2021). This investigation indicates that intermediate-sized species exhibit the most significant shape variation, while small and large cell sizes tend to be predominantly compact (spherical or cubic). Among the various shapes investigated, the authors reported that 46% are considered prolate, 38% compact, and 16% oblate (Ryabov et al., 2021). Prolate shapes are elongated cells with an aspect ratio greater than 1, moving primarily along their longest axis (like a cigar). Oblate shapes are flattened cells with an aspect ratio less than 1, typically moving with their flat face leading, perpendicular to their shortest axis (like a lentil). Compact shapes have an aspect ratio close to 1 and show no strong preference in movement direction. A complicated yet valuable endeavor involves applying the theoretical framework pioneered by (Guo et al., 2021) to calculate the external power dissipation during swimming for these 38 shapes (Ryabov et al., 2021).

Swimming internal efficiency refers to the effectiveness of the internal propulsive layer, axoneme, of the phytoplankton swimmer. The primary sources of energy dissipation in the internal propulsive layer are flagellum bending, sliding of microtubules, and internal viscosity (Spagnolie & Lauga, 2010; Chen et al., 2015). Experiments on the ATP consumption rate per beat cycle of the flagellum in *Chlamydomonas reinhardtii* reveal that the energy dissipation due to the elastic bending of the axoneme is an order of magnitude greater than the internal hydrodynamic energy dissipation (Chen et al., 2015). Chen et al. (Chen et al., 2015) refer to the external efficiency as hydrodynamic efficiency $\left({\text{ε}}_{\text{hydro}}\right)$ and the internal efficiency as chemomechanical efficiency $\left({\text{ε}}_{\text{chemo}}\right)$. They conclude that the overall swimming efficiency $\left({\text{ε}}_{\text{swim}}\right)$ of sea urchin sperm is the product of these two efficiencies, ranging from ${\text{ε}}_{\text{swim}}=0.34\times 0.004=0.001$ to ${\text{ε}}_{\text{swim}}=0.6\times 0.013=0.008$, depending on the viscosity.

The detailed swimming internal efficiency of *Synechococcus* and *Synechocystis* cyanobacteria remains less explored. However, it is evident that any energy dissipation linked with motility mechanisms should be taken into account. For instance, in *Synechococcus*, components such as the S-layer and protein motors moving along helical paths contribute to energy dissipation, while in *Synechocystis*, the type IV pilus could play a similar role. In *Synechococcus* cyanobacteria, the subunits of the S-layer form a two-dimensional lattice with varying spacing, typically ranging from 5 to 22 nm, resulting in oblique, triangular, square, or hexagonal shapes (Šmarda et al., 2002). These structural variations, unique to each species, may lead to different ranges of energy dissipation. This dissipation likely occurs as motor proteins make frictional contact with the S-layer and move beneath it along a helical path, generating traveling waves on the organism's surface (Ehlers & Oster, 2012). Since these motor proteins are driven by proton motive force (Ehlers & Oster, 2012), another source of dissipation likely occurs in this process, presumably in the form of proton leakage, as extensively reported in the membrane of other organelles like mitochondria (Jastroch et al., 2010; Divakaruni & Brand, 2011; Bertholet et al., 2022). In type IV pilus machines in *Synechocystis*, one reported source of inefficiency is related to the retraction process, mostly in the absence of ATPase (Ng et al., 2016; Craig et al., 2019).

One might question the significance of swimming costs in cyanobacteria, given their typically small size and slow swimming speeds - although some species, such as those in the order *Nostocales*, can reach millimeter scales. According to Stokes' law, this would suggest that their swimming incurs minimal energetic cost. However, two important points should be considered: (1) the swimming efficiency of cyanobacteria remains unknown, and (2) the energetic cost of swimming is a relative measure - it must be evaluated in relation to the organism's metabolic rate. Since smaller organisms tend to have lower metabolic rates, even small swimming costs could represent a substantial fraction of their energy budget. The same principle applies to the cost of buoyancy regulation in cyanobacteria, which is explored in the following section.

As briefly discussed in Section 2, some phytoplankton swimmers exhibit jumping-like behavior either to escape predators or to enhance nutrient uptake. However, these jumps come at a high energetic cost. First, according to Stokes’ law, if an organism increases its speed fivefold during a jump, the power required for swimming rises by at least 25 times. Second, additional energy is lost through dissipative processes during each jump. Using computational fluid dynamics (CFD), (Jiang, 2011) demonstrated that although a typical jump lasts only 18.6 milliseconds, it can consume between 8% and 185% of the respiration rate of the photosynthetic *Mesodinium rubrum*, depending on the ciliary arrangement and forcing scheme. Nevertheless, Jiang concluded that if only one jump occurs per second, the average energetic cost of jumping over one second would be relatively small compared to the respiration rate (Jiang, 2011).

#### 5.2.4. Sinking/Rising External and Internal Efficiency

The shape-dependent external efficiency of sinking species appears to be more complex than that of swimmers (Durante et al., 2019). Non-spherical sinkers (both single cells and colonies) generally exhibit a slower sinking rate compared to their equivalent spherical counterparts, except for tear-drop-shaped sinkers (Padisák et al., 2003; Naselli-Flores, Zohary & Padisák, 2021). To the best of our knowledge, there is no solid theory in the literature explaining why the shape-related external efficiency differs between swimmers and sinkers.

The correlation among shape, speed, ocean depth, nutrient concentration, and light availability is significant. For instance, deep waters often exhibit low-light and high-nutrient conditions concurrently. There is an augmentation in cell elongation with increasing depth, regardless of whether the waters are poorly mixed or well-mixed (Ryabov et al., 2021). This is presumably because phytoplankton with elongated shapes are adept at arranging chloroplasts along their cell surfaces, thereby enhancing light absorption, particularly at greater depths (O’Farrell, Pinto & Izaguirre, 2007). The geometric shape is also influenced by seasonal variations, with different species of diatoms exhibiting a predominantly oblate shape in spring and autumn and a predominantly prolate shape in summer (Kléparski et al., 2022).

The internal efficiency of sinking and rising in phytoplankton, such as diatoms, remains largely unknown. This is likely because these species have traditionally been considered passive motile microorganisms in physical models. We propose classifying their motility as active, albeit non-visible. Using this new classification, it is essential to define, calculate, and formulate the internal efficiency of phytoplankton's sinking and rising movements. This internal efficiency can be defined as any dissipation occurring in contact with the components that facilitate sinking/rising. The buoyancy regulation strategy can be more energy-intensive than swimming, owing to the diverse modulatory processes a cell must undertake to move both downward and upward, involving the construction of heavier or lighter macromolecules depending on the condition (Lavoie, Raven & Levasseur, 2016; Raven & Lavoie, 2021). According to Raven and Lavoie (Raven & Lavoie, 2021), the energy cost of synthesizing gas vesicles in cyanobacteria is 1.3 × 10⁻^3^ relative to the total synthesis cost of the cell, whereas the active water influx in the diatom *Ethmodiscus rex* is 0.016 relative to the same.

The major mechanisms facilitating buoyancy regulation in phytoplankton and the possible associated internal energy dissipation are proposed in the following.

1. Gas vesicles, which mediate buoyancy regulation in prokaryotes such as cyanobacteria and haloarchaea, consist of a gas-filled space enclosed by a protein wall primarily composed of proteins like GvpA and GvpC (Walsby, 1994; Pfeifer, 2012; Huber et al., 2023). The efficiency of gas vesicles in providing buoyancy is proposed to be influenced by the interaction of their geometrical properties, elastic modulus, and turgor or hydrostatic pressure (Walsby & Bleything, 1988). This efficiency is qualitatively defined by the volumetric ratio of the gas space, $V_{i}$, to the surrounding proteins, $V_{w}$, which indicates how much buoyant force is generated relative to the structural material (Walsby & Bleything, 1988). As the radius and length of the cylindrical-shaped vesicle increase, the efficiency improves because a larger gas volume provides more buoyancy with relatively less protein weight. However, this improvement in efficiency eventually saturates with increasing length (Walsby & Bleything, 1988; Walsby, 1994). The shape of gas vesicles changes over time, starting as small biconical structures at formation and developing into cylindrical and spindle shapes at maturity (Pfeifer, 2012). Under normal conditions, gas vesicles can achieve efficiencies as high as 90% of the theoretical maximum, defined as $\frac{{\left(\frac{V_{i}}{V_{w}}\right)}_{\text{measured}}}{{\left(\frac{V_{i}}{V_{w}}\right)}_{\text{theor.max}.}}\times 100$, formulated for infinitely long gas vesicles (Walsby & Bleything, 1988). However, changes in hydrostatic pressure, such as those experienced in deep ocean environments or under high light intensity, can reduce this efficiency and may cause gas vesicles to collapse, disrupting buoyancy regulation in cyanobacteria (Abeynayaka, Asaeda & Kaneko, 2017). If the cell survives, reforming gas vesicles is a resource-intensive process (Abeynayaka et al., 2017). The ability of gas vesicles to withstand pressure depends on the elastic modulus of their structure, necessitating a mechanical analysis of stress and strain (Walsby, 1991).
2. Buoyancy regulation in diatoms is managed by balancing protoplasm density adjustments and the silicification process in the frustule. The mass density in both the protoplast and frustule can be either increased or decreased relative to water. Optimal buoyancy regulation occurs when these two density adjustments align positively, meaning both compartments either simultaneously increase or simultaneously decrease their density relative to water. Conversely, if one compartment increases density while the other decreases it, this misalignment reduces efficiency and increases energy dissipation. The density of protoplasm is changed through the processes like the synthesis of organic osmolytes and trimethyl ammonium (Boyd & Gradmann, 2002; Lavoie et al., 2016; Raven & Lavoie, 2021), and the active influx of water from the cytosol into the contractile vacuole (Raven & Doblin, 2014; Lavoie & Raven, 2020) while the density of frustule is changed through silicification (Finkel & Kotrc, 2010; van Tol, Irwin & Finkel, 2012). The silicification process generally leads to an increase in frustule density (Raven & Waite, 2004). However, it has been reported that ocean acidification can significantly reduce silica production (Petrou et al., 2019). Additionally, bacterial attack on diatoms using their hydrolytic enzymes increases the rate of silica dissolution, resulting in a lower mass density of diatoms (Patrick & Holding, 1985; Bidle & Azam, 1999). Other possible sources of dissipation might involve cell expansion, which leads to mechanical deformation and the release of elastic energy during mass density changes. This process is also associated with the relative movement of the valves of the frustule, potentially contributing to hydrodynamic dissipation. Given the rigidity of the frustule and the average force required to break it (0.2 – 0.8 mN (Friedrichs et al., 2013)), any deformation can be energetically costly. The leakage of solutes through the membranes (tonoplast and plasmalemma), associated with changes in the mass density of the vacuole and the cell wall, is another source of energy dissipation (Raven, 1987).
3. Calcification associated with the calcified shells (coccospheres) of coccolithophores significantly alters their sinking rates. For instance, in small *E. huxleyi*, which ranges from 4 to 9 μm in size, calcification changes the sinking speed from 3 cm/d to 30 cm/d - an order of magnitude difference between naked and calcifying organisms (Monteiro et al., 2016). While the literature primarily examines the role of calcification in increasing mass density and sinking rates in coccolithophores (Hoffmann et al., 2015; Monteiro et al., 2016), these organisms can also regulate buoyancy by stopping calcification under extreme light-limited conditions, thereby lowering cell density (Young, 1987; Rost & Riebesell, 2004). Under nutrient-limited conditions, such as phosphate or nitrogen deficiency, cell growth is restricted, limiting the use of absorbed light energy for biosynthesis. However, calcification continues despite its energy demand, serving as a mechanism to dissipate excess absorbed light energy that cannot be utilized for growth. This energy dissipation helps protect the cell from potential photodamage (Paasche, 2001; Rost & Riebesell, 2004). Possible sources of hydrodynamic dissipation may also exist within the cell-wall layer, involving the interplay of coccoliths, body scales, and baseplate scales during the calcification process and density adjustment. The coccosphere has multiple roles beyond buoyancy regulation, including mechanical protection, as evidenced by its strong inelastic tolerance to forces (Jaya et al., 2016), protection against photodamage (Amirian et al., 2024), and acceleration of photosynthesis (Monteiro et al., 2016). These diverse roles complicate the assessment of the cost of calcification associated with internal buoyancy-related mechanisms. One approach to assess the role of calcification in buoyancy regulation is to measure the oxygen consumption rate under dark conditions when the phytoplankton is both suspended and sinking. This can be achieved using optical fluorescence oxygen sensors (Katsu-Kimura et al., 2009) or modern methods that measure extracellular acidification and oxygen consumption rate, providing ATP turnover within the cell (Mookerjee et al., 2017).

The dynamic sinking behavior observed in large diatoms - characterized by bursts of rapid downward motion lasting a few seconds followed by brief pauses - has been reported in studies by (Gemmell et al., 2016; Du Clos et al., 2021). While this unsteady vertical motion enhances nutrient flux, it may also incur a non-negligible energetic cost (Lavoie & Raven, 2020). Lavoie and Raven have estimated that the minimum energy expenditure for this behavior accounts for 16% of the total energy cost of growth. This is achieved through the modulation of sodium and potassium permeability, interconversion of low-density and high-density organic cations, and alterations in cell expansion rate (Lavoie & Raven, 2020). The rapidly fluctuating processes are recognized for their high energy dissipation rates in nonequilibrium thermodynamics (Blaber, Louwerse & Sivak, 2021), a factor that should be considered in future analyses. We suggest that this unsteady sinking behavior can be mathematically simulated by incorporating a stochastic term into the buoyancy equation (Equations (2) or (3)). This stochastic term, representing a time-dependent ‘burst noise,’ should be defined by a base noise level, a burst amplitude, and a random component, and should be multiplied by the organism's mass density.

To adjust its internal density, an organism needs to change its mass and volume so that their ratio increases (to move downward) or decreases (to move upward) relative to the density of water. A rapid and sudden change in mass and volume is analogous to the inflation and deflation of a balloon. Classical physics shows that the first attempt to increase the balloon's radius requires much more pressure than subsequent inflations (Ješková, Featonby & Feková, 2012; Vandermarlière, 2016). This is due to the resistance of the polymer chains' chemical bonds in the balloon until a threshold stretch is reached (Stein, 1958). Similarly, in species that exhibit rapid bursts of speed when sinking or rising, the initial sudden effort to increase/decrease the mass density and consequently the speed may dissipate much more energy compared to typical slow adjustments of mass density. This hypothesis could be tested in future experimental studies.

#### 5.2.5. Predator and Virus Risk

In this section, the question is how predation and viral risks *directly* or *indirectly* affect the cost of motility.

- The direct cost arises from the increased speed required for evasion, pursuit, or escape, leading to higher motility power consumption as described by Stokes' law.
- Indirect costs happen when the organism must spend part of its energy on defense, such as making toxins or other protective measures. This reduces the energy available for other functions. As a result, even if the swimming cost stays the same, it becomes a larger fraction of the total energy budget.

Theoretical models for dinoflagellates show that producing toxins can significantly reduce the rate of cell division especially under nitrogen-starved conditions (Chakraborty et al., 2019; Kiørboe, 2024). These toxins are generally produced to deter grazers or competitors, providing a defense mechanism despite the cost to growth. Producing saxitoxin requires at least 132 moles of ATP to synthesize one mole of the substance (Chakraborty et al., 2019). The number of moles of saxitoxin per cell can be determined based on the cell’s nitrogen content. Assuming 10^−11^ g N per cell, with 15% devoted to toxin production (Chakraborty et al., 2019), and given the molecular formula of saxitoxin (C_10_H_17_N_7_O_4_), where the nitrogen content per mole of saxitoxin is 7 × 14 g, we calculate: (0.15 × 10^−11^) / (7 × 14) = 1.5 × 10^−14^ mol of saxitoxin per cell. Multiplying by 132 moles of ATP per mole of saxitoxin gives 2.0 × 10^−12^ mol ATP per cell, which, when converted using Avogadro’s number, results in 1.2 × 10^12^ ATP molecules per cell. Under nutrient-limited conditions, this significantly reduces the cell-division rate by more than 20% (Chakraborty et al., 2019).

Prey can utilize other defense mechanisms, such as the production of blue bioluminescence, which can startle or deter predators and thus reduce the risk of being eaten (Haddock, Moline & Case, 2010). This process incurs a high energetic cost due to the chemical reaction between luciferin substrate and luciferase enzyme in the presence of oxygen (Valiadi & Iglesias-Rodriguez, 2013; Cusick & Widder, 2020) which requires approximately 60 ATP molecules per photon (Hastings, 1978; Cusick & Widder, 2020). Each bioluminescent flash can last 0.1 to 0.5 s and can involve the production of 10^7^ to 10^11^ photons per second (Marcinko et al., 2013), equating to a minimum of 60 × 0.1 × 10^7^ = 6 × 10^7^ to a maximum of 60 × 0.5 × 10^11^ = 3 × 10^12^ ATP molecules per flash.

Defense costs may be lower owing to the inherent cell-wall structure of the prey. For instance, copepods efficiently hunt thin-shelled diatoms, yet they frequently reject thick-shelled ones (Ryderheim, Grønning & Kiørboe, 2022).

Phytoplankton may be infected by viruses, which can alter their sinking speed. The sinking speed of infected phytoplankton, e.g., in *H. akashiwo*, may increase as a defense mechanism to remove viruses attached to their surface and may also increase due to a rise in the mass density of the host cell (Lawrence & Suttle, 2004; Danovaro et al., 2011). Studies on flagellated bacteria have shown that, while swimming may increase susceptibility to viral infection, hydrodynamic interactions mitigate this risk around the cell body but exacerbate it near the flagellar bundle. This is a consequence of differences in fluid flow dynamics around the flagella compared to those around the cell body (Lohrmann, Holm & Datta, 2024). Experiments with ciliates fed various viruses, including mammalian viruses and bacteriophages, indicate that the presence of certain phages progressively increases the swimming speed of ciliates. This increase in swimming speed is interpreted as a behavioral response, suggesting that *Tetrahymena pyriformis* senses these viruses and recognizes them as potential food, similar to its response to nonviral prey (Olive et al., 2022). However, the costs and benefits associated with viral infection on the swimming or sinking behavior of phytoplankton remain unknown. Table 2 provides a summary of all the motility costs discussed in this paper.

### 5.3. Motility Cost to Total Metabolic Rate Ratio

To determine what percentage of the total metabolic rate of the cell is accounted for by the motility mechanisms, one requires data on the total metabolic rate of the studied species. This task is more straightforward for heterotrophic protists under growing or starved conditions, as it can be obtained through the rate of oxygen consumption (Fenchel & Finlay, 1983; Crawford, 1992; DeLong et al., 2010). However, this poses a greater challenge for phytoplankton cells, whether they are photoautotrophs or mixotrophs, because these microorganisms are both producers of oxygen in their chloroplasts and consumers of oxygen in their mitochondria and cytoplasm (Kliphuis et al., 2011). ATP is widely regarded as the universal energy currency of most living systems (Nicholls & Ferguson, 2013), with both constructional and operational costs typically expressed in terms of ATP molecules (Lynch, 2024; Schavemaker & Lynch, 2025).

We evaluate the potential costs associated with phytoplankton motility using the dinoflagellate *Prorocentrum mariae-lebouriae* as an example. This species is currently considered a synonym of *Prorocentrum minimum* (Pavillard) J.Schiller (Tomas, 1997). *Prorocentrum minimum* is a geographically widespread, bloom-forming dinoflagellate linked to harmful algal blooms, ecosystem damage, and potential shellfish toxicity, often associated with coastal eutrophication (Heil, Glibert & Fan, 2005). This dinoflagellate measures 14.8 μm in length, 14.8 μm in width, 7.3 μm in depth, and 5.85 μm in equivalent spherical radius, with an oblate shape and an aspect ratio of 0.49 (Kamykowski, Reed & Kirkpatrick, 1992). It has a volume (𝑉) of 846 μm^3^ and a mass density of 1064 kg/m^3^, resulting in a total body mass of approximately 0.9 ng and a swimming speed of 170 μm/s at 20°C (Kamykowski et al., 1992).

The construction cost of flagella ($C_{\text{f}}$ [#ATP]) can be estimated using a fitting equation provided by (Schavemaker & Lynch, 2022):

(9). $$C_{\text{f}}=2.6\times 10^{10}V^{0.31}$$

This yields a cost of approximately 2.1×10^11^ ATP molecules, or 17.4 nJ, for the dinoflagellate under study. As described in previous sections, the length of the flagella can vary by up to ±20% due to fluctuations in the removal and addition of amino acids and proteins. Consequently, the construction cost ranges approximately from 13.9 nJ to 20.8 nJ. The construction cost of flagella should be evaluated in relation to the total construction cost of the entire cell. We assume that the cell's construction cost is determined by the total energy content of all its constituent proteins, carbohydrates, and lipids. A dinoflagellate with a volume of 846 μm^3^ has a total chemical energy content $E\left[\frac{\text{nJ}}{\text{cell}}\right]$ (comprising proteins, carbohydrates, and lipids) of 5.3×10^3^ nJ/cell. This value is derived using the equation developed for dinoflagellates (Finkel, Follows & Irwin, 2016):

(10). $$\log_{10}\;E=2.88+0.91\;\log_{10}\left(\frac{V}{100}\right)$$

Thus, the construction cost of the flagella ranges from 0.26% to 0.39% of the total construction cost of the *P. minimum* body.

Next, we estimate the operational cost as a fraction of the organism’s total metabolic rate. Based on Stokes' law (Equation 1), the minimum power required for the organism to tow its spherical body can be estimated as $P=6\pi \eta rv^{2}$. However, as discussed in this review, several corrections to this equation must be considered.

1. The average microscale viscosity at the cell surface of plankton is approximately 2.6 times the viscosity of seawater, as reported in (Guadayol et al., 2021). The bulk viscosity of seawater at 20°C is 0.00109 [kg.m^−1^s^−1^].
2. For external efficiency related to the physical properties of flagella, such as the flagellum-to-body length ratio, and slenderness of the flagellum, we consider the average efficiency reported for green algae (Tam & Hosoi, 2011; Barsanti et al., 2016) to be 1%. This indicates that for every 100 units of energy, 99 units are dissipated, meaning the organism must expend 99 times more energy to compensate for the energy required for swimming. Consequently, the swimming minimum power consumption estimated by Stokes’ law should be multiplied by 100.
3. In the above calculation of external efficiency, the organism’s body is approximated as a rigid sphere (Tam & Hosoi, 2011; Barsanti et al., 2016). However, the dinoflagellate examined in our example has an aspect ratio of 0.49. Using the relationship between swimming external efficiency and body aspect ratio developed by (Nasouri et al., 2021), the aspect ratio of 0.49 for *P. minimum* yields an external efficiency of 0.16. This indicates that its oblate body shape results in lower efficiency compared to a spherical body, which has an efficiency of 0.33 (a simple way to obtain this value is by fitting a function to the plot in Figure 3 of (Nasouri et al., 2021)). Therefore, the operational cost should be multiplied by approximately 2.1.
4. Next, we consider the elastic cost of bending the axoneme, which is associated with swimming internal efficiency (Spagnolie & Lauga, 2010; Chen et al., 2015). As reported by (Chen et al., 2015), the chemomechanical efficiency (referred to in our article as internal efficiency) of sea urchin sperm ranges from 34% to 60%, depending on the viscosity. We use a representative value of 50%, meaning that the operational cost should be multiplied by 2.
5. We do not include the cost of jumping, as it varies between species (and not all species exhibit jumping behavior). Additionally, accurate estimation requires knowledge of the frequency and duration of jumps.
6. In this estimation, we assumed the dinoflagellate relies solely on swimming without buoyancy regulation. However, since dinoflagellates can perform both, if they do so simultaneously, the molecular costs and internal efficiency of mass density adjustment would factor into the above calculations, potentially increasing the overall motility cost.

Therefore, the power consumed during swimming can be calculated as follows:

$$\begin{array}{l} P_{\text{tot}}=\left(6\pi \eta rv^{2}\times 100\times 2.1\times 2\right)\text{pW}\\ \;=6\pi \times 2.6\times 0.00109\left[\text{kg}\cdot {\text{m}}^{-1}{\text{s}}^{-1}\right]\times 5.85\times 10^{-6}[\text{m}]\times {\left(170\times 10^{-6}\right)}^{2}\left[{\text{m}}^{2}{\text{s}}^{-2}\right]\times 100\times 2.1\times 2\times 10^{12}\\ \;\approx 3.8\;\text{pW}. \end{array}$$

To assess whether this operational cost is significant, it should be compared to the organism’s total metabolic rate - that is, its overall rate of ATP production.

A precise assessment of the total ATP production rate in phytoplankton requires knowledge of the respiration rate (Tang, 1995), mitochondrial proton leak (Brand et al., 1999; Jastroch et al., 2010), ATP yield per oxygen molecule consumed in mitochondria (Brand, 2005), photosynthesis rate (Geider & Osborne, 1989; Langdon, 1993), proton leak across the thylakoid membrane of the chloroplast (Richter et al., 2004; Buchert, Bailleul & Joliot, 2021), and the total ATP yield per oxygen molecule produced in the chloroplast, accounting for non-cyclic, cyclic, and pseudo-cyclic electron transport pathways (Allen, 2002; Burlacot, 2023). However, such a detailed assessment is beyond the scope of the present work. Therefore, we provide a rough estimate to illustrate how the total available power content per organism can be approximated.

One might question that ATP produced in chloroplasts is entirely consumed by carbon fixation and other intra-chloroplast processes, so why should the cost of motility be evaluated relative to the total energy produced by both mitochondria and chloroplasts? Mitochondria and chloroplasts function in a tightly interconnected manner. For example, in diatoms, chloroplasts generate oxygen during photosynthesis, which can be utilized by mitochondria for respiration in addition to oxygen absorbed from the surrounding environment (Bailleul et al., 2015). In turn, mitochondria supplement chloroplasts by producing and supplying ATP when photosynthetic ATP production is insufficient to meet the chloroplasts’ energy demands (Bailleul et al., 2015). This energetic interplay is supported by a dynamic exchange of reducing equivalents (electron carriers) between the two organelles, underscoring their close metabolic interdependence and coordinated functioning (Bailleul et al., 2015). Given this interdependence, we propose that motility cost should be evaluated relative to the combined energy production from both mitochondria and chloroplasts.

The respiration rate in phytoplankton - based on a general relationship not specific to dinoflagellates - is scaled with cell volume according to the equation (López-Sandoval et al., 2014):

(11), logR=0.9logV−4.1

where R is the respiration rate in pmol O_2_ cell^−1^ h^−1^, V is the cell volume in μm^3^, and the equation applies at 18°C under exponential growth. Assuming that each mL of O_2_ yields approximately 20 J of energy (Fenchel & Finlay, 1983), the respiration rate for *P. minimum*, with a cell volume of 846 μm^3^, corresponds to 4.27 pW. Adjusting this to 20°C using a Q_10_ of 2.5 increases the value to 5.13 pW. The photosynthesis rate in phytoplankton under exponential growth follows the equation (López-Sandoval et al., 2014):

(12), logP=0.91logV−2.14

where P is the photosynthesis rate in pg C cell^−1^ h^−1^, also at 18°C. Assuming an energy content of 39 kJ g^−1^ C (Kleidon, 2021), the photosynthesis rate for *P. minimum* yields approximately 36.2 pW. Since temperature has a much smaller effect on photosynthesis than on respiration specifically under nutrient replete conditions (Marañón et al., 2018), we do not apply a temperature correction to photosynthesis rate. Summing respiration and photosynthesis rates gives a total metabolic power of roughly 41.3 pW for *P. minimum* at 20°C. Thus, the previously estimated operational cost of 3.8 pW represents about 10% of the organism’s total metabolic rate - a substantial proportion. We emphasize that this is an average value, and the actual cost may be higher or lower depending on specific metabolic conditions. This relative cost reflects the intrinsic energetic burden associated with active relocation in this species. This cost is likely to increase under nutrient or light limitation during migration.

Dinoflagellates actively respond to turbulent conditions using various strategies, including density adjustment, shape modification, and changes in orientation (Sengupta et al., 2017). These dynamic changes affect swimming power consumption, as they are directly related to both speed and the swimming internal and external efficiencies. The density changes in dinoflagellates are proposed to be mediated by the cell nucleus, with a mass density of 1300 kg/m^3^, and by 15–20 chloroplasts, each with a mass density of 1150 kg/m^3^ (Sengupta et al., 2017). The actual costs associated with these rapid changes under turbulence remain unknown, representing a gap in our knowledge. However, it has been observed that the rate of stress, measured as the rate of production of the nitric oxide (NO) free radical under turbulent conditions, increases (Sengupta et al., 2017). Estimating the energetic cost of NO production and its accumulation in the cell constitutes the second gap for future studies, even though the mechanisms involved in the production of this radical in living systems are known (Burov et al., 2022).

## 6. Temperature-Dependent Costs of Motility

The projections regarding the future of climate change and global warming raise several concerns, one of which revolves around the fate of marine phytoplankton and their role in carbon cycling within the ocean (Doney, 2006; Regaudie-De-Gioux & Duarte, 2012; Sekerci & Petrovskii, 2015; Barton et al., 2016; Heneghan et al., 2021; McGinty et al., 2021; Lotze et al., 2022; Sheward et al., 2023; Mutshinda, Finkel & Irwin, 2025). Establishing a mechanistic model that links motility costs to temperature variations appears complicated, given the myriad of effective parameters discussed in previous sections.

In a recent study (Svensson, Norberg & Snoeijs, 2014) on phytoplankton behavior, researchers proposed that movement towards warmer locations, rather than nutrient availability, was the primary driver for the development or maintenance of motility in these organisms. This is because higher temperatures increase metabolic rates, enhancing the energy available for movement, while simultaneously lowering water viscosity, making locomotion energetically cheaper. Additionally, warming intensifies stratification by creating stronger temperature gradients in the water column, which limits vertical mixing and leads to nutrient-depleted surface layers. Under these conditions, motility becomes a crucial adaptive trait, enabling organisms to actively navigate between nutrient-rich deeper layers and light-rich surface waters. According to (Svensson et al., 2014), seasonal temperature variations did not significantly influence motility in benthic diatoms, suggesting that the effects of sustained warming differ fundamentally from natural seasonal changes.

The effect of ocean acidification on motility in aquatic organisms appears to be context-dependent, varying by species, cell type, and duration of exposure. In the harmful algal species *Heterosigma akashiwo*, both short-term and generational-scale exposure to elevated CO_2_ (low pH) led to enhanced motility - particularly increased downward swimming speed. In contrast, studies on coral and sea cucumber sperm (Morita et al., 2010; Nakamura & Morita, 2012), and flagellated algal species (*Microglena sp.*, *Dunaliella salina*, and *Chlamydomonas reinhardtii*) (Wang et al., 2020) demonstrated that acidification impairs motility. Notably, the long-term (5-year) exposure study revealed a conserved reduction in motility across diverse taxa, suggesting that elevated CO_2_ may broadly compromise the flagellar machinery across eukaryotic lineages (Wang et al., 2020). On top of these temperature and CO_2_ effects, evolutionary and community composition changes can further influence motility patterns over time.

Stokes' law (Equation (1)) provides the idea that estimating the temperature-dependent cost of motility requires knowledge of how viscosity, cell size, and motility speed vary with temperature. When combined with the temperature dependence of the organism's metabolic rate, these factors can be used to estimate the relative cost of motility across temperature gradients. However, even this simplified scenario is inherently complex, and the actual situation is even more so. We begin by focusing on the temperature dependence of viscosity, cell size, motility speed, and metabolic rate. We then address other indirect effects of temperature on the cost of motility.

Temperature affects both the viscosity and density of seawater, which in turn influences hydrodynamic forces. As temperature increases, water becomes less viscous and less dense, reducing the resistance experienced by objects moving through it. These changes modify the Reynolds number, leading to variations in drag and inertial forces (Escobar et al., 2016).

Research on the temperature dependence of the size of bacteria, protists, and metazoa indicates a general trend of decreasing species size linearly (at both the single-cell and colony levels) with rising temperatures (Atkinson et al., 2003; Zohary, Flaim & Sommer, 2021). Temperature is indeed recognized as a major factor influencing size structure, both directly and indirectly (Zohary et al., 2021). Although no definitive general theory has been established for the temperature-size rule, several hypotheses have been proposed to explain this phenomenon: First, as temperature rises, the availability of essential resources such as carbon dioxide and dissolved oxygen decreases, while metabolic demand increases. Smaller cells can more effectively absorb and utilize these limited resources due to their higher surface area-to-volume ratio, thereby compensating for the reduced resource availability (Atkinson et al., 2003). Contrary to this hypothesis, some bacteria do not depend on gases for survival. Additionally, nutrient availability is not directly temperature-dependent, as it varies by geographical location - some regions become depleted around 20°C, while others do so near 0°C (Kamykowski et al., 2002). Second, smaller cell size may facilitate faster reproduction, enhancing survival and competitive advantage (Atkinson et al., 2003). However, this raises the question: why donť organisms remain small in size permanently? Contrary to these propositions, Maranon et al. (Marañón et al., 2012) have reported that there is no overarching law elucidating the impact of temperature on phytoplankton size structure. They emphasize that resource availability serves as a major factor influencing size structure. An example that contradicts Atkinson’s size-temperature rule is the finding that *Thalassiosira pseudonana* diatoms increase in size with rising temperatures in evolved lineages after 300 generations of adaptation (Schaum et al., 2018). This size increase is hypothesized to enhance lipid content, serving as a strategy to mitigate oxidative stress induced by high temperatures (Leles & Levine, 2023).

Beveridge et al. (Beveridge et al., 2010) introduced a model to describe different scenarios involving the temperature dependency of protists' swimming speed, influenced by the interplay between temperature-dependent viscous drag and metabolic rate. The formula they have derived for swimming speed integrates Stokes' law, mass-specific metabolic rate, and the temperature dependencies of viscosity and size, expressed as follows:

(13), $$v=\sqrt{\frac{a_{0}i_{0}{\left[M(T)\right]}^{3/4}{\text{e}}^{-E/k_{B}T}}{6\pi \eta (T)r(T)}}$$

where $a_{0}$ represents the proportion of metabolic rate allocated to swimming, $i_{0}$ is a normalization constant with the units kg^1/4^.m^2^.s^−3^, M(T) is the temperature dependence of body mass, derived from the assumption of a 2.5% decrease in cell size per 1°C temperature increase in protists (Atkinson et al., 2003), $k_{B}$ is the Boltzmann constant, and E is the activation energy. Their findings suggest that swimming speed escalates with rising temperatures (Beveridge et al., 2010), aligning with other research exploring temperature-dependent sinking speeds (Bach et al., 2012; Naselli-Flores et al., 2021). However, Kamykowski and McCollum's investigation of dinoflagellates' temperature-acclimatized swimming speed across 5–38°C under stable nutrient and light conditions reveals a trend where speed increases with temperature until reaching a threshold, beyond which it declines (Kamykowski & Mccollum, 1986). They report that the temperature range supporting swimming ability is broader than the range permitting growth (Kamykowski & Mccollum, 1986). In their study, the maximum swimming speed is observed in cells approximately 35 μm in length. This decline in speed at higher temperatures and larger sizes can be attributed to physiological limitations and inefficiencies in motility machinery - factors not accounted for by the Arrhenius equation.

To capture how total metabolic rate (i.e., ATP production rate) responds to temperature, one should consider several underlying parameters at play. Respiration rate, for instance, typically follows a Q10 between 2 and 3, averaging around 2.5 (Hansen, Bjørnsen & Hansen, 1997). Proton leakage increases with temperature, causing a portion of the metabolic energy to be lost as heat, as demonstrated in mitochondria from the eurythermal bivalve *Mya arenaria* (Abele et al., 2002). The ATP yield per molecular oxygen consumed in mitochondria also shows temperature dependence, as seen in intact mitochondria of *Arabidopsis thaliana* (Kerbler, Taylor & Millar, 2019). This is because the electron transport chain (ETC) is less sensitive to temperature changes than ATP synthase (Kerbler et al., 2019). Temperature also influences both the catalytic activity and abundance of ATP synthase in plant mitochondria (Tan, Millar & Taylor, 2012). Furthermore, the respiration-to-photosynthesis ratio shifts with temperature due to differing thermal sensitivities of respiration and photosynthesis, as observed in *Chlorella vulgaris* (Padfield et al., 2016). Under nutrient-limited conditions, the activation energy of photosynthesis can be up to three times that of respiration, as shown in *Emiliania huxleyi*, *Skeletonema costatum*, and *Synechococcus* species (Marañón et al., 2018).

In the following, we present two examples illustrating how temperature influences the motility machinery (in this case, flagella) and its efficiency, concluding this section by highlighting the challenges involved in modeling the temperature-dependent costs of motility.

Assembly and function of flagella (Huang et al., 1977), the ATP-hydrolysis rate by molecular motors involved in motility (Yadav & Kunwar, 2021), and the internal viscosity and density, are all temperature-dependent, influencing internal efficiency in swimming or sinking. Moreover, temperature-dependent changes in surface tension (Lee et al., 2001; Kranenburg & Smit, 2005), elasticity (Waugh & Evans, 1979; Kranenburg & Smit, 2005; Kirsch & Böckmann, 2019), and permeability (Fang et al., 2017) of the cell membrane result in alterations in cell shape and size, thereby affecting external efficiency in swimming or sinking.

Researchers studied how temperature affects the flagella of the algal species *Chlamydomonas reinhardtii* (Huang et al., 1977). They found that when the temperature shifts from 20°C to 32°C, the flagella respond differently depending on the specific mutant being observed. This range of responses includes flagella becoming broken down, detached, or malfunctioning, all of which incur energetic costs (Huang et al., 1977). Additionally, the model proposed in (Humphries, 2013) shows that the temperature dependence of beat frequency of eukaryotic cilia and that of the angular velocity of bacterial flagella are governed by the relationships:

(14), $$f=\sqrt{c_{1}Te^{-T\left(x_{1}+x_{2}\right)}+c_{2}}-c_{3}$$

(15), $$\omega =\sqrt{c_{4}T-c_{5}}e^{-\frac{T\left(x_{1}+x_{2}\right)}{2}}+c_{6}$$

where T represents absolute temperature, f denotes the beating frequency, ω is the angular velocity, $x_{1}$ and $x_{2}$ are temperature-viscosity fitting parameters of the fluid and the cell respectively, and $c_{1,\ldots ,6}$ are fitting constants related to parameters including the amplitude, wavelength, and ATP concentration.

## 7. Coupling of Phytoplankton Size and Motility Speed

Using Stokes’ law to estimate the operational cost of motility raises the question of whether the parameters in the equation are interrelated. One important potential coupling is whether swimming or sinking speed varies with organism size. Recent studies (Lisicki et al., 2019; Lynch & Trickovic, 2020; Nielsen & Kiørboe, 2021; Schavemaker & Lynch, 2022; Kamdar et al., 2023) consistently suggest that swimming speed of unicellular eukaryotes shows either no dependency on cell volume or a weak sub-linear dependence. However, some older references (e.g. (Kamykowski et al., 1992)) propose that intermediate-sized dinoflagellates display the highest swimming speeds.

A challenge encountered in the analyzed data across the literature is the limited sample size, which often fails to encompass the entire spectrum of volume and speed ranges. Additionally, data collected from different studies covering a wide array of sizes and speeds may vary in terms of temperature, physiological conditions, and other influencing parameters. Hence, the initial approach to addressing this issue involves acquiring a comprehensive and uniform dataset that spans across all size and speed ranges ideally normalized to temperature. A similar situation applies to the sinking speed of species. Generally, it is reported that the sinking rate increases linearly with cell size (Durante et al., 2019; Cael, Cavan & Britten, 2021), but this relationship is not considered universal due to the high uncertainties between the scattered empirical data and the theoretical models.

**Figure 2:**
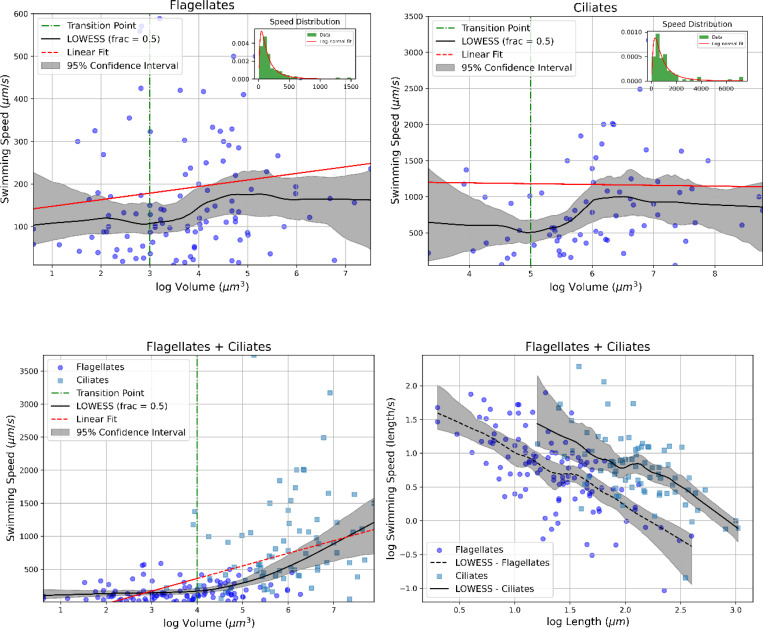

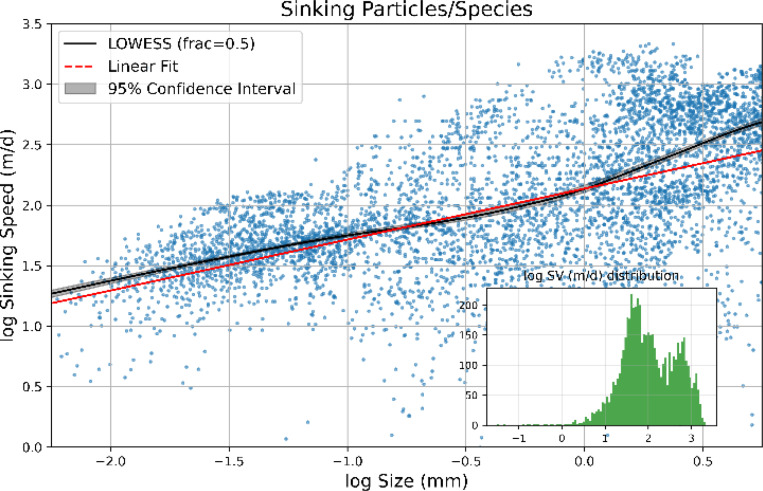
LOWESS regression curves depicting the semi-log volume[μm^3^]-speed[μm/s] relationship for flagellates (top-left), ciliates (top-right), a combination of flagellates and ciliates (middle-left), log-log length[μm]-speed[length/s] relationship for both flagellates and ciliates (middle-right), and the log-log size[mm]-speed[m/d] plot of sinking species/particles (bottom).

One strategy for dealing with highly scattered data involves identifying nonlinear patterns through smoothing techniques. One such method is Locally Weighted Scatterplot Smoothing (LOWESS) (Simonoff, 2012), which segments the dataset into subregions, conducts linear regressions within each subregion, and then connects them using a cubic function. Here, we apply this approach to analyze the swimming speed versus size data of 111 flagellates and 78 ciliates collected by (Lisicki et al., 2019) from different references, along with data on sinking speed of marine particles and living species versus size obtained from 5650 sinkers collected in (Cael et al., 2021). The data on flagellates and ciliates follow a log-normal distribution (Lisicki et al., 2019), whereas the log-transformed data of sinking particles and species exhibit a bimodal distribution with negative skewness according to our analysis. The result is shown in Figure 2.

When should these nonlinearities be considered in data analysis? Nonlinear data analysis is necessary when nonlinear regression is statistically significant with a considerable confidence interval and when the observed nonlinearity, along with its associated variations and noise, has a meaningful physiological interpretation.

In Figure 2, none of the nonlinearities are significant except in the middle-left panel, which shows a transition in speed across species from flagellates to ciliates. In Figure 2, the semi-log plots in the top row - flagellates (upper left) and ciliates (upper right) - do not demonstrate a statistically significant nonlinear or linear pattern. However, the steepest slopes of the LOWESS curves in flagellates and ciliates considerably greater than the slopes of the corresponding linear regressions specifically in ciliates. Combining data for flagellates and ciliates (middle-left) reveals a statistically significant increase in swimming speed (μm/s) for log volumes (μm^3^) greater than 4 (vertical green line), attributable to taxonomic differences. This suggests an average increase in speed during the transition from flagellates to ciliates. The middle-right figure represents the log-log plot of swimming speed (body-lengths per second) versus body length (μm) for both flagellates and ciliates. As shown, swimming speed relative to body length decreases with increasing cell size in both groups, exhibiting similar slopes. However, this analysis represents speed-to-length ratio versus length (a spurious correlation of ratios), meaning that since both axes include length, it may introduce a statistical artifact rather than capturing a true biological trend. Given the lack of clear linear or nonlinear patterns in the top-left and top-right figures, along with substantial noise in the data, the observed decreasing trend and similar slopes between flagellates and ciliates in the middle-right figure could be an artifact of division bias. Therefore, this trend requires further investigation in future studies. In sinking species/particles (bottom, log-log plot), the LOWESS curve diverges from linear regression for log sizes (in mm) exceeding zero with a high confidence interval. Based on this analysis, we cannot yet confirm or refute the hypothesis of a relationship between size and speed in phytoplankton swimmers and sinkers.

We also analyzed the dataset compiled by (Velho Rodrigues, Lisicki & Lauga, 2021), which includes measurements of body length and swimming speed for bacteria, archaea, flagellates, ciliates, and spermatozoa. Our findings align with those discussed in Figure 2. The only distinct pattern is observed in spermatozoa, which show a slight negative correlation between swimming speed and body length, suggesting that larger sperm tend to swim more slowly. However, this correlation is weak.

## 8. Conclusion

1. This work presents a comprehensive re-evaluation of phytoplankton motility, challenging the traditional dichotomy between “active” swimming and “passive” sinking. By integrating biological, physical, and ecological evidence, we propose that both swimming and buoyancy-regulated movement involve substantial energy costs. The overarching picture that emerges is that motility in phytoplankton is not a binary trait but a continuum of energetically demanding strategies shaped by physiological and environmental trade-offs. These costs - often underestimated or overlooked - must be balanced against survival benefits such as nutrient acquisition, predator avoidance, and successful migration through heterogeneous marine environments. This perspective reframes our understanding of phytoplankton behavior and physiology, offering an energetic basis for explaining ecological patterns such as the paradox of slower-growing, yet more motile, species like dinoflagellates.
2. We are still some distance away from achieving a comprehensive cost-benefit analysis of phytoplankton swimming and sinking behaviors, with numerous uncertainties remaining. Some less-explored topics encompass the cost-benefit analysis of dynamic strategies employed in response to migratory or turbulent conditions, such as sudden bursts of movement, alterations in shape and orientation, fluctuations in mass density, formation of chains, subdivision into subpopulations, viral infections, depth-dependent environmental and physiological changes, temperature dependent motility variations, and viscosity gradients.
3. We synthesized diverse aspects of the costs and benefits of phytoplankton motility, ranging from the level of a single cell to the scale of the entire ocean. Our goal was to achieve an integrative understanding of this phenomenon by utilizing the current knowledge available in the literature and proposing new avenues for future investigations. We conducted a thorough examination of the motility costs of a model dinoflagellate, estimating these costs relative to the organism's available energy resources.
4. We find that the efficiency and construction costs associated with sinking/rising-related machinery, as well as the swimming mechanisms of surface-distorted cyanobacteria, remain largely unexplored. Phytoplankton sinkers/risers are active, motile microorganisms with invisible buoyancy regulation mechanisms. We investigated the construction costs and potential sources of internal energy dissipation related to motility in diatoms, cyanobacteria, and coccolithophores. This can aid biophysicists in developing equations to model the internal efficiency of buoyancy-regulating phytoplankton.
5. The physics underlying the shape-related external efficiency of sinkers remains an open problem. Specifically, why do spherical sinkers achieve higher speeds compared to sinkers with other geometric shapes, while prolate-shaped swimmers exhibit higher speeds than swimmers with spherical or oblate shapes?
6. Oblate-shaped phytoplankton swimmers with an aspect ratio of ~0.01 exhibit remarkably high swimming external energy dissipation, up to 10^6^ times that of prolate-shaped microswimmers with an aspect ratio close to 100, making swimming a highly inefficient strategy. Why do these species possess swimming abilities despite such inefficiencies? For example, in bacteria, flagella serve multiple functions beyond swimming, including mechanosensing (Belas, 2014), wetness sensing (Anderson, Smith & Hoover, 2010), adhesion (Moens & Vanderleyden, 1996), and contributing to virulence (Moens & Vanderleyden, 1996).
7. It is still early to conclude whether there is a clear relationship between motility speed and the size of plankton species. This remains a gap in the literature, pending the achievement of coherent datasets that cover the full range of microorganism sizes and speeds, all in stable physiological and environmental conditions. Such datasets may need to be collected on a phylum-by-phylum, genus-by-genus, or species-by-species basis.

## Figures and Tables

**Table 1:** The flow velocity equations modeled for various phyla of flagellated phytoplankton (Nielsen & Kiørboe, 2021), are generated from time-averaged point forces with magnitude F applied at a source point. These equations describe the flow velocity at a field point (x,y,z) in Cartesian coordinates, where $d=\sqrt{x^{2}+y^{2}+z^{2}}$ represents the distance between the origin and the field point. The chrysophyte studied in (Nielsen & Kiørboe, 2021) is a sessile cell that generates a feeding current using a single beating flagellum (see Movie S2 in (Nielsen & Kiørboe, 2021)). This beating flagellum can be modeled as a point force acting at the origin, directed along the z-axis in 3D space, with x, y, and z representing the spatial coordinates of the beating flagellum. The resulting velocity field resembles that of a stokeslet, which is a fundamental solution to the Stokes equation in free space (without boundaries) (Pozrikidis, 1992). In the velocity field equation, η represents the viscosity of the fluid. The raphidophyte studied in (Nielsen & Kiørboe, 2021) propels itself by generating force at the front of its body through the motion of its flagella (see Movie S1 in (Nielsen & Kiørboe, 2021)). In these organisms, the flagellar beat is typically oriented so that the flagellum pulls the organism forward through the surrounding fluid, rather than pushing it from behind (meaning the force applied by the flagellum is opposite to the force applied by the cell body). For the derivation of the equation, refer to the supporting information in (Kiørboe et al., 2014). The haptophyte and chlorophyte studied in (Nielsen & Kiørboe, 2021) are assumed to propel themselves using a three-stokeslet quadrupole point force model, where two or more flagella generate, mainly, lateral propulsive forces of equal magnitude, pushing the flow backward, while the opposing drag force of the moving cell body balances the sum of these propulsive forces (see Movie S4 in (Nielsen & Kiørboe, 2021)). The flow velocity field equation is developed in (Andersen et al., 2015).

Phylum	Flagellar arrangement	Point force model	Equation	Ref.
Chrysophyte	One beating flagellum	Stokeslet: A single point force	$v=\frac{F}{8\pi \eta }\frac{\sqrt{x^{2}+y^{2}+4z^{2}}}{d^{2}}$	Supplementary information in (Nielsen & Kiørboe, 2021).
Raphidophyte	One beating flagellum + Opposing force of the cell body	Puller stresslet: Two opposing equal point forces at positions (x=0,y=0,z=±b)	$v=\frac{Fb}{4\pi \eta }\frac{\left|x^{2}+y^{2}-2z^{2}\right|}{d^{4}}$	
Haptophyte Chlorophyte	One or more lateral flagella + Opposing force of the cell body	Three-stokeslet quadrupole: Two opposing equal point forces at positions (x=±a,y=0,z=0) and a third, doubled, point force applied at the origin	$v=\frac{Fa^{2}}{8\pi \eta }f(d)$ *	

* $f(d)=\left(\sqrt{4x^{4}\left(x^{2}+21z^{2}\right)-3x^{2}\left(y^{2}+z^{2}\right)\left(y^{2}+13z^{2}\right)+{\left(y^{2}+z^{2}\right)}^{2}\left(y^{2}+16z^{2}\right)}\right)/d^{6}$

**Table 2:** Summary of motility costs for various swimmers and buoyancy-regulating organisms, including flagellated phytoplankton, cyanobacteria, diatoms, and coccolithophores.

**Phytoplankton Motility Costs**	Swimming Machineries	Construction costs	- Flagella synthesis, remodeling, and degradation (genetically-controlled length fluctuations, and loss rates). - Crystalline surface layer, SwmA glycoproteins, SwmB polypeptides, and peptidoglycan-associated proteins. - Type IV pilus (T4P) machines.	
		Operational costs	External efficiency	- Geometric shape of the cell body. - Length, number, geometry, angle, and arrangement of flagella
			Internal efficiency	- Elastic bending of the axoneme. - Sliding of microtubules. - Internal viscosity. - Hydrodynamic dissipation caused by motor protein movement generating surface waves. - Proton leak. - Retraction process. - Jumping behavior
	Buoyancy Regulating Machineries	Construction costs	- Synthesis of gas vesicles and organic osmolytes. - Channels mediating water influx/efflux in vacuoles. - Proteins responsible for ion exchange of heavy and light ions in vacuoles. - Frustule formation. - Formation of coccolith layers.	
		Operational costs	External efficiency	- Geometric shape of the cell body.
			Internal efficiency	- Ratio of gas space to protein wall volume in gas vesicles. - Turgor or hydrostatic pressure - Elastic modulus of gas vesicles. - Density change alignment between protoplasm and frustule. - Cell expansion (elastic energy of morphological changes). - Hydrodynamic dissipation. - Solutes leakage. - Rapid bursts of movements. - Continuous calcification under nutrient-limited conditions.
Other Costs (for both swimmers & sinkers)	Direct costs: - Increased speed due to viruses/predator risk, and turbulence. - Environmental high local viscosity gradients. - Navigation costs. - Reorientation and change in motion direction requiring dynamic body shape adjustments, mass density modifications, and alterations in center of mass and buoyancy, which impact both external and internal motility efficiency. - Chaotic path. Indirect costs: - Defense mechanisms, along with nutrient and light limitations during migration, reduce total metabolic power, leading to relatively higher motility costs.			
